# Drug resistance profiling of a new triple negative breast cancer patient-derived xenograft model

**DOI:** 10.1186/s12885-019-5401-2

**Published:** 2019-03-07

**Authors:** Margarite D. Matossian, Hope E. Burks, Steven Elliott, Van T. Hoang, Annie C. Bowles, Rachel A. Sabol, Bahia Wahba, Muralidharan Anbalagan, Brian Rowan, Mohamed E. Abazeed, Bruce A. Bunnell, Krzysztof Moroz, Lucio Miele, Lyndsay V. Rhodes, Steven D. Jones, Elizabeth C. Martin, Bridgette M. Collins-Burow, Matthew E. Burow

**Affiliations:** 1 Department of Medicine, Section of Hematology & Medical Oncology, Tulane University School of Medicine, New Orleans, LA USA; 2 Department of Pharmacology, Tulane University School of Medicine, New Orleans, LA USA; 3 Tulane Center for Stem Cell Research and Regenerative Medicine, New Orleans, LA USA; 4 Department of Structural and Cellular Biology, Tulane University, New Orleans, LA USA; 5 Cleveland Clinic, Department of Radiation Oncology, Cleveland, OH USA; 6 Tulane Cancer Center, New Orleans, LA USA; 7 Department of Pathology, Tulane University School of Medicine, New Orleans, LA USA; 8 Department of Surgery, Tulane University School of Medicine, New Orleans, LA USA; 9 Louisiana Cancer Research Center, Biospecimen Core, New Orleans, LA USA; 10 Department of Genetics and Stanley S. Scott Cancer Center, Louisiana State University Health Sciences Center, New Orleans, LA USA; 11 Department of Biology, Florida Gulf Coast University, Fort Myers, FL USA; 12 Department of Agricultural Engineering, Louisiana State University, Baton Rouge, LA USA

**Keywords:** Triple-negative breast cancer, Patient-derived xenograft, Mammosphere, Chemoresistance

## Abstract

**Background:**

Triple-negative breast cancer (TNBC) represents an aggressive subtype with limited therapeutic options. Experimental preclinical models that recapitulate their tumors of origin can accelerate target identification, thereby potentially improving therapeutic efficacy. Patient-derived xenografts (PDXs), due to their genomic and transcriptomic fidelity to the tumors from which they are derived, are poised to improve the preclinical testing of drug-target combinations in translational models. Despite the previous development of breast and TNBC PDX models, those derived from patients with demonstrated health-disparities are lacking.

**Methods:**

We use an aggressive TNBC PDX model propagated in SCID/Beige mice that was established from an African-American woman, TU-BcX-2 K1, and assess its metastatic potential and drug sensitivities under distinct in vitro conditions. Cellular derivatives of the primary tumor or the PDX were grown in 2D culture conditions or grown in mammospheres 3D culture. Flow cytometry and fluorescence staining was used to quantify cancer stem cell-like populations. qRT-PCR was used to describe the mesenchymal gene signature of the tumor. The sensitivity of TU-BcX-2 K1-derived cells to anti-neoplastic oncology drugs was compared in adherent cells and mammospheres. Drug response was evaluated using a live/dead staining kit and crystal violet staining.

**Results:**

TU-BcX-2 K1 has a low propensity for metastasis, reflects a mesenchymal state, and contains a large burden of cancer stem cells. We show that TU-BcX-2 K1 cells have differential responses to cytotoxic and targeted therapies in 2D compared to 3D culture conditions insofar as several drug classes conferred sensitivity in 2D but not in 3D culture, or cells grown as mammospheres.

**Conclusions:**

Here we introduce a new TNBC PDX model and demonstrate the differences in evaluating drug sensitivity in adherent cells compared to mammosphere, or suspension, culture.

**Electronic supplementary material:**

The online version of this article (10.1186/s12885-019-5401-2) contains supplementary material, which is available to authorized users.

## Background

Novel therapeutic target discoveries and the recent focus on precision medicine have dramatically advanced oncology therapeutic research. Clinical trials with targeted inhibitors in oncology have high failure rates [1, 2]; in fact, only 7.5% of the oncology drugs that entered phase I clinical development were approved for clinical use (pooled data from the US, Europe and Japan). Furthermore, only 33.2% of drugs that entered phase III trials are approved for clinical use and over 90% of phase 3 clinical trials in oncology fail to meet their primary endpoints. These high failure rates highlight the need for better predictive preclinical models [3]. The reason for these failure rates is multifactorial, but one of the major contributing factors is the limitations of current preclinical models used [4]. Triple negative breast cancer (TNBC) is a clinically aggressive, molecularly heterogeneous group of malignancies that currently has no clinically approved small molecule targeted therapies. There are currently no clinically approved small molecule targeted agents or immunotherapy agents for TNBC [5]. TNBC has a propensity to metastasize, recur, and develop chemoresistance, making it a notoriously difficult disease to treat. Moreover, patients have a considerably worse overall survival [6, 7]. TNBC lacks expression of receptors (estrogen, HER2/Neu amplification) that are targeted by commonly used therapies, either endocrine-targeting or HER2 + -targeting. More predictive preclinical models are required to adequately evaluate therapeutic response in the laboratory setting. While no preclinical model can definitively predict responses to cancer therapeutics, patient-derived xenograft (PDX) models recapitulate many of the complex components of the patient tumors and are emerging as important preclinical tools in therapeutic discovery.

PDX models are patient tumor tissue (primary or metastatic lesions) directly implanted in immunocompromised mice; in our studies we use severe combined immunodeficient (SCID)/Beige mice to propagate the tissue and preserve viability. If the tissue is kept intact during implantation, many of the features present in the original tumor in the patient are maintained in early passage murine models: the tumor cells, stromal components, extracellular matrix (ECM) composition and the 3-dimensional (3D) architecture of the ECM components within the tumor [8]. All these aspects of a tumor are unique to each patient, promoting the potential of PDX models in the discovery of personalized therapeutics [9]. In fact, not only are these models crucial to predicting drug responses in the preclinical setting in solid tumors [10], but also have been used to predict clinical response of chemotherapies for individual breast cancer patients [9, 11].

PDX-derived cells in preclinical therapeutic studies represent more translational models compared to immortalized, established cell lines. Although cell lines have provided valuable insight into mechanisms driving cancer progression, they do not address molecular and genomic features unique to individual patients’ tumor cells. Chemosensitivity differs from patient to patient, even with identical clinical and pathological presentations [12]. Cell line-based experiments, especially in adherent culture conditions (also referred to in the remainder of the manuscript as ‘two-dimensional or 2D’) are easy to manipulate and set up for large scale screens in therapeutic discovery research. Certain drug classes, however, cannot be evaluated properly by adherent cell screening experiments, including signal transduction inhibitors, antibodies, bioreductive drugs, antiangiogenic peptides or small molecules and anti-telomerases [13]. Furthermore, growth in adherent conditions acquires mutations and genetic/epigenetic changes that are not present in the original tumor from which the cells were derived [14]. Response to oncology drugs also fails over time as tumor cells are grown in adherent conditions [15] and observations reported in adherent culture do not translate into clinical observations [16]. One example of this phenomena is that while Erlotinib, an epidermal growth factor receptor (EGFR) -targeted inhibitor, enhanced radiosensitivity in 2D cells, there was no efficacy in phase II clinical trials [17]. Due to increased discrepancies in translation of therapies, as observed with Erlotinib, 3D cell culture systems are being utilized to replace or complement 2D cultures as the preferred technique for drug screens [18–20]. Low-attachment conditions more accurately represent physiologic behaviors of the cancer cells. Cell adherence to plastic or glass surfaces forces cells to grow in monolayers, and inhibits cells from forming multi-dimensional structures, as cells would behave in native in vivo tissues [21]. Additionally, 3D culture models are relatively easy to multiplex for medium-throughput screening purposes.

We first introduce a new TNBC PDX model, TU-BcX-2 K1, and we describe baseline characteristics of this model. Then we use TU-BcX-2 K1-derived cells to demonstrate the importance of using 3D cell culture conditions in addition to 2D, or adherent, culture conditions, in evaluating chemosensitivity in in vitro studies. For these experiments, we use a live/dead fluorescence stain; this technique not only provides information regarding the efficacy of the drugs in individual patient’s cancer cells, but also facilitates discovery of new mechanisms of specific drugs which can be pursued in future experiments. This is especially important in examining the mechanisms of targeted small molecule inhibitors in TNBC.

## Materials and methods

### Reagents

Dulbecco’s modified Eagle’s medium (DMEM), Dulbecco’s phosphate-buffered saline (DPBS), phenol-red free DMEM, fetal bovine serum (FBS), minimal essential amino acids, non-essential amino acids, antibiotic/anti-mitotic, sodium pyruvate, ethylenediaminetetraacetic acid (EDTA 0.5 M, pH 8) and trypan blue were obtained from GIBCO (Invitrogen; Carlsbad CA). Insulin was purchased from Sigma-Aldrich (St. Louis MO). Dimethyl sulfoxide (DMSO) was obtained from Research Organics, Inc. (Cleveland OH).

### Adherent cell culture

From a molecular standpoint, TU-BcX-2 K1 cells had gene expression profiles closest to the Mesenchymal (M) subtype described by Lehmann et al. [22, 23]. Adherent/2D cells were maintained in DMEM supplemented with 10% FBS, non-essential amino acids, essential amino acids, anti-anti (100 U/mL), sodium pyruvate and porcine insulin (1 × 10^− 10^ mol/L) at 37 °C in humidified 5% CO_2._

### Patient-derived xenografts

The triple negative patient-derived tumor, designated as TU-BCx-2 K1, was acquired in collaboration with the Louisiana Cancer Research Consortium Biospecimen Core and were processed in compliance with NIH regulations and institutional guidelines, and approved by the Institutional Review Board at Tulane University. The TU-BcX-2 K1 model was derived from a biopsy specimen from an African-American patient who had not yet initiated a chemotherapy regimen. For comparison of tumor growth, we also mention TU-BcX-2O0, a claudin-low TNBC PDX model established from an African American patient; TU-BcX-2O0 was also treatment naïve. TU-BCx-2 K1 was established and propagated in immunocompromised SCID/Beige mice. SCID/Beige *(*CB17.Cg-*Prkdc*^*scid*^*Lyst* ^*bg*^/Crl*)* 4–6 weeks of age were purchased from Charles River and were used to prevent rejection of the xenografted human tumors. The autosomal recessive SCID (Prkdc^scid^) mutation results in severe combined immunodeficiency affecting both the B and T lymphocytes. The autosomal recessive beige (Lyst^bg^) mutation results in defective natural killer (NK) cells. Mice acclimated for 5–7 days in sterile laboratory cages with appropriate bedding material and autoclaved food and water before experiments were performed. Tumor tissues from patients were dissected into 3 × 3 mm^3^ pieces under aseptic sterile conditions, coated with full factor Matrigel™ (Cat No. 354234, Fisher Scientific, Waltham, MA, USA) and implanted bilaterally into the mammary fat pads (MFPs) of SCID/Beige mice under isoflurane and oxygen. When tumors were large enough to be palpable, tumors were measured using a digital caliper. When tumor volume reached 750–1000 mm^3^, tumors were passaged, or serially transplanted, into new mice. For serial transplantation, the mice with the large PDX tumors were euthanized by CO_2_ and cervical dislocation, and tumors were removed, dissected to 3 × 3 mm^3^ pieces and coated in full factor Matrigel™. The coated tumors were then implanted bilaterally into new mice that were anesthetized using a mix of isoflurane and oxygen delivered by mask. Before surgery, mice were given Meloxicam (5 mg/kg/day, for 3 days post-surgery) for pain and mice were monitored for 3 days for evidence of distress; if distress was observed, mice were euthanized. For ex vivo analysis, TU-BCx-2 K1 explants were collected, and RNA was extracted using enzymatic digestions using QIAzol Lysis Reagent (Cat No. 79306; Qiagen, Valencia, CA, USA) and mechanical disruption using scissors. Total RNA was isolated, and cDNA was synthesized using the iScript cDNA synthesis kit (Bio-Rad, Hercules, CA). cDNA was analyzed with quantitative reverse transcription polymerase chain reaction (qRT-PCR). Primers (Invitrogen, Carlsbad, CA) were generated with sequences as follows: *β-actin* F-5′- GGCACCCAGCACAATGAAGA-3′; *β-actin* R-5′- ACTCCTGCTTGCTGATCCAC -3′; *CDH1* F-5′-AGGTGACAGAGCCTCTGGATAGA-3′, *CDH1* R-3′-TGGATGACACAGCGTGAGAGA-3′; *CDH2* F-5′-GCCCCTCAAGTGTTACCTCAA-3′, *CDH2* R-5′- AGCCGAGTGATGGTCCAATTT-3′; *VIM* F-5′-CGTCCACCCGCACCTACAGC-3′, *VIM* R-5′-GCCAGCGAGAAGTCCACCGAG-3′; *CD24* F-5′- TGCTCCTACCCACGCAGATT-3′, *CD24* R-5′- GGCCAACCCAGAGTTGGAA-3′; *cFOS* F-5′- GAATGCGACCAACCTTGTGC-3′; *cFOS* R-5′- AGGGATCAGACAGAGGGTGT-3′; *SNAI1* F-5′- AGCCGTGCCTTCGCTGACC-3′; *SNAI1* R-5′- GGACTCTTGGTGCTTGTGGAGC-3′; *FRA1* F-5′- CGAAGGCCTTGTGAACAGAT-3′, *FRA1*-R-5′- CTGCAGCCCAGATTTCTCA-3′; *TWIST* F-5′- TGTCCGCGTCCCACTAGC-3′ *TWIST* R-5′- TGTCCATTTTCTCCTTCTCTGGA-3′; *SLUG* F-5′- TGTTGCAGTGAGGGCAAGAA-3′, *SLUG* R-5′- GACCCTGGTTGCTTCAAGGA-3′; *cMYC* F-5′- CAGCGGGCGGGCACTTTG-3′, *cMYC* R-5′- AGAGAAGCGGGTCCTGGCA-3′. qRT-PCR was conducted as previously published [24]. Data represented as normalized fold expression compared with DMSO control of biological triplicate samples ± S.E.M.

### Establishment of TU-BCx-2 K1 cell line

A TU-BcX-2 K1 tumor piece (3 × 3 mm^2^) was plated in a 6-well plate with DMEM supplemented with 10% FBS, non-essential amino acids (NEAA), MEM amino acids, anti-anti (100 U/mL), sodium pyruvate and porcine insulin (1 × 10^− 10^ mol/L) at 37 °C in humidified 5% CO_2._ TU-BCx-2 K1 was generated from cells that adhered to the dish weeks after the explant was plated.

### Mammosphere culture

Mammospheres were cultured in low-attachment (also referred to as 3D culture) in DMEM/F-12 media supplemented with B-27, penicillin-streptomycin, fibroblast growth factor (FGF) and epidermal growth factor (EGF) (Invitrogen, Carlsbad, CA) at 37 °C in humidified 5% CO_2_. Mammospheres were created by plating TU-BCx-2 K1 PDX cells (50,000 cells) in low suspension DMEM/F-12 media supplemented with fibroblast and epidermal growth factors (20 ng/mL each; Miltenyi Biotec, Bergisch Gladbach, Germany) in low-attachment 6-well plates (ThermoFisher Scientific, Waltham MA). Growth factors were added to the spheres every 3 days. Sphere growth was observed with brightfield microscopy and representative images were captured every 3 days.

### Immunohistochemical staining

Tumors were fixed in 10% buffered formalin for 24 to 36 h. Paraffin-embedded sections (4 μm thickness) mounted on slides were manually deparaffinized in xylene, rehydrated in a series of graded ethanol solutions, steamed in Diva Decloaker (Antigen retrieval solution, Biocare Medical) for 30 min for antigen retrieval before 5-min incubation with 3% hydrogen peroxide to block endogenous peroxidase. Sections were washed with PBS, blocked for 30 min in 10% normal goat serum (Invitrogen), and incubated overnight in primary antibody (CDH1, Cell Signaling Technologies 3195S; 1:400). After incubation with primary antibody, slides were rinsed in PBS, incubated with biotinylated secondary antibody (Vector labs) for 30 min, washed with PBS followed by incubation with ABC reagent (Vector labs) for 30 min. Staining was visualized through incubation in 3, 3-diaminobenzidine and counterstaining with Harris hematoxylin. As negative control, samples were incubated with either 10% goat serum or non-specific rabbit IgG. After dehydration, slides were mounted with Permount (Fisher) and visualized using a Nikon OPTIPHOT microscope. Bright-field images (200X magnification) were captured by Nikon Digital Sight High-Definition color camera (DS-Fi1) using NIS-Elements BR software.

### Live/dead fluorescence stain

TU-BcX-2 K1 cells were plated in 96-well plates in either adherent (2D) culture conditions or low-attachment (3D) culture conditions at 2000 cells per well. After 24 h, cells were treated with the National Cancer Institute (NCI) oncology drug panel (https://dtp.cancer.gov/organization/dscb/obtaining/available_plates.htm). Adherent cells were treated for 3 days, low-attachment cells were treated for 5 days. Cells were washed with phosphate buffered saline and stained with a mixture of Calcein-AM (2 μM) and Ethidium homodimer (EthD)-III (5 μM) purchased from PromoKine (New York, USA; Cat. No. PK-CA707–30002). Stained cells were imaged with confocal microscopy and images were captured (8 images per well of adherent cells, 5 images per well of low-suspension cells). The 588 nM excitation channel was used to identify red, ‘dead’ cells, and the 420 nM excitation channel was used to visualize green, ‘live’ cells. ApoTome (commercial structure illumination microscopy by Zeiss, Thornwood, NY) fluorescent images were captured on an inverted microscope (Zeiss). Representative images were taken at 100x magnification. Dead and live cells were quantified using the ImageJ program. The total number of cells remaining after treatment as well as the relative number of live and dead cells were recorded.

### Embedded mammospheres and immunofluorescence staining for CSC markers

For Matrigel™-embedded experiments, TU-BcX-2 K1 primary mammospheres were plated in low-attachment 96-well plates and covered with 40% Matrigel™. For treatment experiments, primary mammospheres were pre-treated with DMSO control, Taxol (10 nM) or romidepsin (100 nM) for 72 h before plating. Spheres were stained in-well with primary conjugated CD44 (FITC anti-mouse/human CD44 antibody, BioLegend, San Diego CA; Cat. No. 103021) and CD24 (PE anti-human CD24 antibody, BioLegend, San Diego CA; Cat. No. 311105), and DAPI nuclear stain (NucBlue Fixed Cell Stain ReadyProbe, Life Technologies, Carlsbad CA). ApoTome (commercial structure illumination microscopy by Zeiss, Thornwood, NY) fluorescent images were captured on an inverted microscope (Zeiss) and digitally filtered to obtain optical slices. Z-stack imaging was captured using confocal microscopy using the Zeiss microscope setup.

### Flow cytometry

When PDX tumors were passaged from one mouse to the next, both the cells within the tumors and circulating cells in the peripheral blood of the PDX-implanted mice were analyzed for immune and CSC markers. TU-BcX-2 K1 tumors were enzymatically digested with type I collagenase (Worthington Biochemical Corporation, Lakewood, NJ, USA) at room temperature, neutralized with media, and then filtered. Circulating tumor cells were collected in whole blood with 0.5 M EDTA (Gibco Invitrogen, Carlsbad CA), incubated in red blood cell lysis buffer (0.008% NH4Cl, pH 7.2–7.4; Sigma-Aldrich, St. Louis MO) and washed with phosphate buffered saline (PBS). Isolated, harvested cells from the tumor and blood samples were placed in staining solution containing 1% Bovine Serum Albumin (Sigma-Aldrich) and 1% CD16/CD32 Mouse BD Fc BlockTM (BD Biosciences) in PBS. Anti-human CD24 (APC), anti-human CD326 (EpCAM; PerCP-eFluor710) and anti-human/mouse CD44 (PE-Fluor 610) primary antibodies were purchased from eBiosciences (San Diego, CA, USA). Cell populations from the tumors and blood were analyzed using a Galios Flow Cytometer (Beckman Coulter, Brea, CA, USA) running Kaluza software (Beckman Coulter). At least 5000 events were analyzed and reported as the mean ± standard error of mean (SEM).

### Crystal violet staining

TU-BcX-2 K1 cells were stained with trypan blue, and viable cells were plated (2000 cells per well) in a 96-well plate in 10% DMEM. Cells were treated with the vehicle or the NCI oncology drug panel for 72 h and the plate was incubated in 37 °C, 5% CO_2_. The plate was then harvested by adding 10 μL glutaraldehyde to each well for 20 min. After rinsing and drying the plate, the cells were stained with 50 μM 0.1% crystal violet in 90% methanol for 20 min. After another rinse, the cells were left overnight to dry, and the following day morphological alterations of the cells were visualized with an inverted microscope and images were recorded. Cells were lysed with 33% acetic acid and quantified to determine proliferation after treatment. The Nikon eclipse TE2000-s inverted fluorescence microscope and camera with x-cite series 120 illuminator (Nikon; Melville, NY), in conjunction with IP Lab version 3.7 software (Rockville, MD) were used in the visualization of crystal violet-treated cells to observe morphological changes.

### Statistical analysis

Studies run in triplicate were analyzed by unpaired Student’s *t-*test (Graph Pad Prism V.4). *p-*Values < 0.05 were considered statistically significant. Data is represented as mean ± SEM. All dose/response, flow cytometry and qRT-PCR experiments are performed in triplicate, unless stated otherwise.

## Results

### Baseline characteristics of TU-BcX-2 K1

**Fig. 1:**
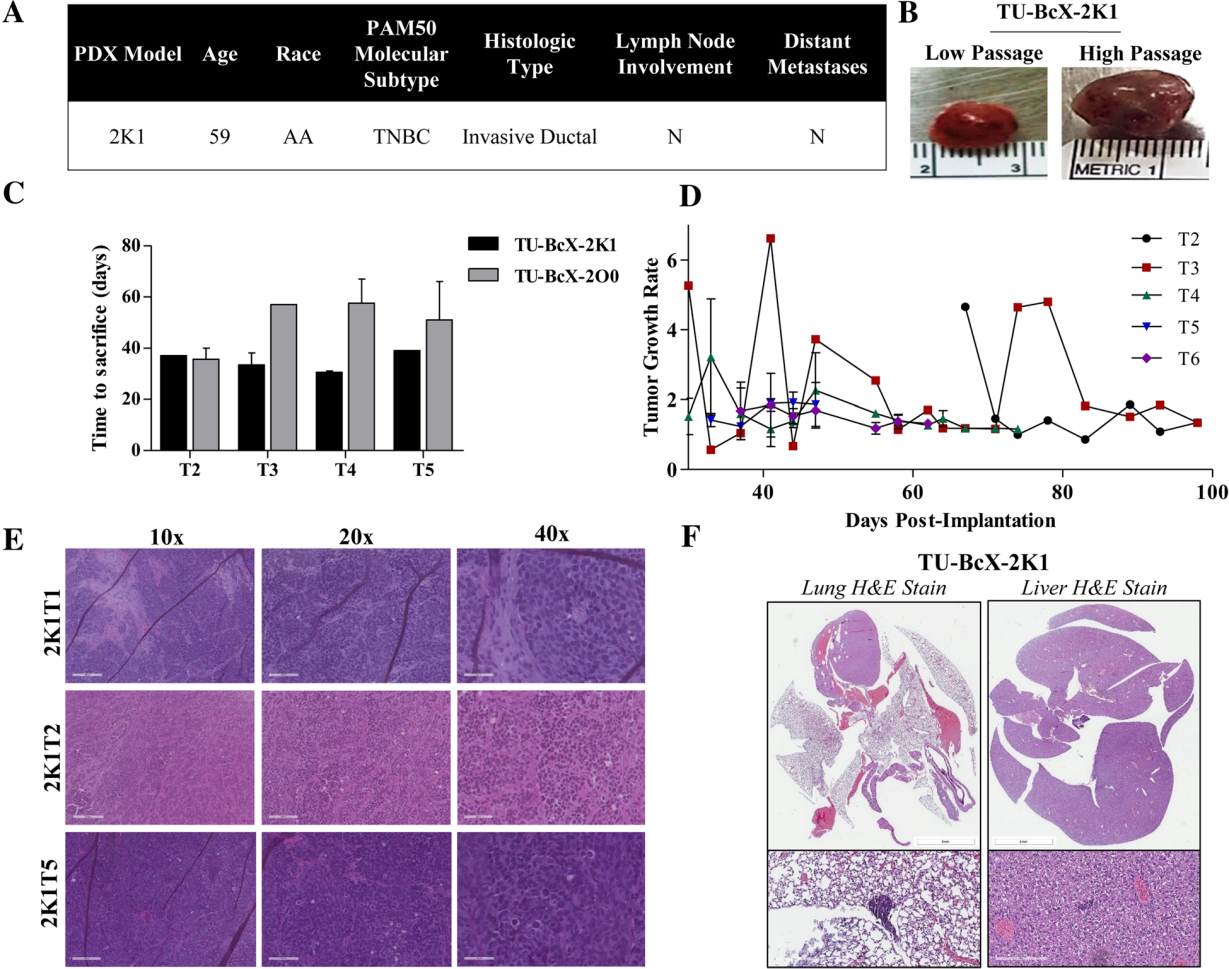
Characterization of TU-BCx-2 K1. **a** TU-BCx-2 K1 was derived from the biopsy specimen of a 59-year-old African-American female. This PDX tumor was categorized as a TNBC PAM50 molecular subtype and was diagnosed as an invasive ductal carcinoma. There was no evidence of lymph node nor distal metastases at the time of resection. **b** Representative gross images of lower and higher passage TU-BCx-2 K1 as well as the tumor thawed from cryopreservation. The tumor was homogenous and solid in cross-section. **c** After implantation into the mammary fat pads of SCID/Beige mice, TU-BCx-2 K1 tumor reached 1000mm^3^ after approximately 40 days. Days to tumor take did not vary significantly throughout various passages. At the end of the PDX model name, ‘T’ denotes the passage of the tumor in mice; for example, ‘2K1T2’ means the tumor was passaged two times in mice before analysis. **d** Comparison of growth rates of tumors implanted in mice at different passages (T2-T6). **e** H & E staining of TU-BCx-2 K1 tumors revealed cells with aberrant mitoses surrounded by areas of fibrosis. This histologic appearance did not change dramatically between passages in mice. **f** Representative H & E images of lungs and livers harvested from mice implanted with TU-BcX-2 K1 (passage 3 in mice). Minimal metastatic lesions found in both lungs and livers. Inserts are shown at 200X magnification

**Fig. 2:**
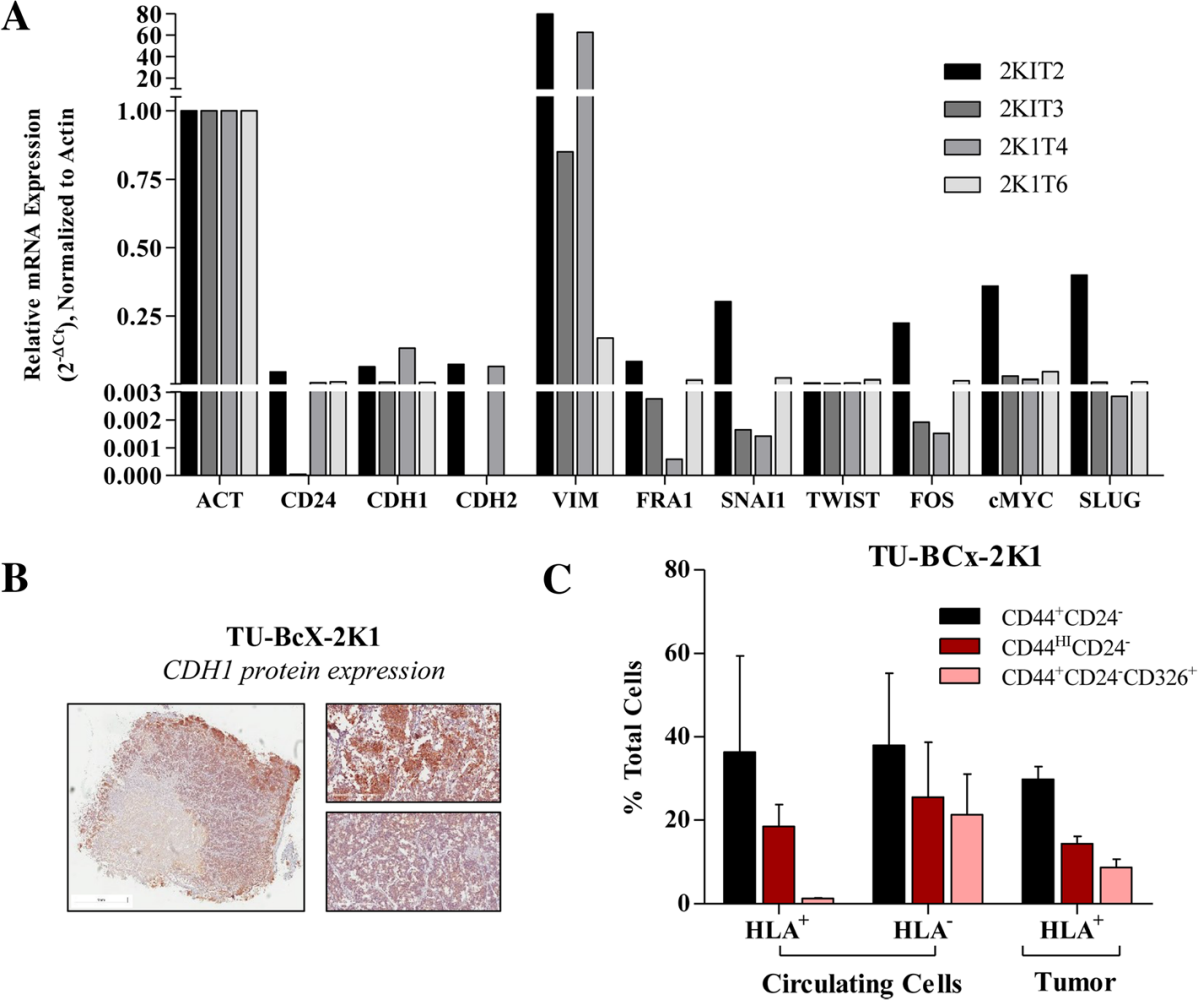
Mesenchymal and cancer stem-cell like features in TU-BcX-2 K1 cells. **a** Panel of epithelial (*CD24, CDH1*) and mesenchymal (*CDH2, VIM, FRA1, SNAI1, TWIST, cFOS, cMYC* and *SLUG*) genes analyzed by qRT-PCR in TU-BcX-2 K1 tumors passaged in mice (T2, T3, T4, T6). There were no endogenous levels of CD44 in any TU-BcX-2 K1 tumors passaged in mice and very low levels of CDH2 in T3 and T6 and CD24 in T3. Due to limited tissue availability, one sample was obtained per passage and analyzed. Data was normalized to β-actin. **b** Immunohistochemistry staining for CDH1 protein expression in the TU-BcX-2 K1 T3 tumor. Representative images in inserts are shown at 200X magnification. **c** Flow cytometry of circulating tumor cells and matched TU-BCx-2 K1 tumor explants. Mouse cells are HLA- and human cells are HLA+; *N* = 2

The TU-BcX-2 K1 TNBC PDX model was derived from the biopsy specimen of a 59-year old African-American female who had not yet initiated a chemotherapy regimen and was confirmed by the Department of Pathology at Tulane University to be a PAM50 TNBC subtype. The histologic type of the tumor was invasive ductal, and there was no involvement of lymph node nor distant metastases present in the patient at the time of biopsy (Fig. 1a). After that initial implantation and tumor take of the biopsy specimen, intact tumor pieces were implanted into the MFPs of immunocompromised SCID/Beige mice for tissue propagation. In gross appearance, TU-BcX-2 K1 is a solid, firm, tan-colored tumor that was homogenous in nature (Fig. 1b). TU-BcX-2 K1 exhibited consistent tumor growth in each passage, taking approximately 40 days in each passage to reach minimum measurable volume (1000mm^3^). For comparison, this was a shorter time between passages compared to another PDX model established at the same time, TU-BcX-2O0 (Fig. 1c). TU-BcX-2O0 is a claudin-low TNBC, treatment naïve PDX model that was also established from an African-American patient. Growth rates of tumors throughout various passages in mice were overall consistent, with the most variability in growth rates in the lower passage, T2 and T3, tumors implanted in mice (Fig. 1d). Various passages of the whole, intact tumor were stained with Hematoxylin & Eosin (H & E) and show cell histology and the presence of fibrosis was preserved throughout consecutive passages (Fig. 1e). Lungs and livers were harvested at the time of each passage and organs were formalin fixed, paraffin-embedded and H & E stained to highlight metastases. TU-BcX-2 K1 forms micrometastatic lesions in the lungs and livers of SCID/Beige mice, although they were minimal. These observations were consistent throughout lower and higher passages (Fig. 1f). TU-BcX-2 K1 represents a mesenchymal phenotype based on the mesenchymal appearance of the TU-BcX-2 K1-derived cells and the gene signatures of both the PDX-derived cells and intact tumor pieces. For both cell line and tumor pieces, evaluation of gene expression demonstrates high endogenous expression of select mesenchymal genes (*VIM, cMYC, FRA1, SNAI1* and *SLUG*), and low endogenous expressions of epithelial gene *CD24* and mesenchymal genes *TWIST* and *ZEB1* (Fig. 2a). Relative mesenchymal gene expression varied with different passages. Notably, *CDH2* mRNA expression was very low in T3 (1.8 × 10^− 6^ relative expression) and T6 passages (7.41 × 10^− 6^ relative expression, and *CD24* expression was low in T3 (3.95 × 10^− 5^ relative expression) normalized to actin. E-cadherin (*CDH1*) expression was present in the TU-BcX-2 K1 tumors on both transcript and protein levels, although *CDH1* expression was localized to the outside of the tumors (Fig. 2b). We next examined the immune cell population within TU-BcX-2 K1 tumors; we found low populations markers of both angiogenesis (CD31) and granulocytes (CD14). Within mouse cell populations (HLA^−^), there was a significantly higher population of CD14^+^ cells, indicating mouse-derived immune cell invasion into the tumor, as would be expected after consecutive passaging in mice (Additional file 1: Figure S1).

Next, to determine if cancer cells derived from the TU-BcX-2 K1 tumor maintained similar characteristics as the primary tumor, the PDX-derived cell line from early passage TU-BCx-2 K1 tumors was established. In adherent culture conditions, TU-BcX-2 K1 derived cells were mesenchymal in phenotype, defined as spindle-shaped with cellular protrusions, minimal cell-cell contact and fibroblastic-like. TU-BcX-2 K1-derived cells were capable of forming spheres in 3D culture conditions (Additional file 2: Figure S2). To further evaluate the sphere-forming capabilities of TU-BcX-2 K1, we first performed flow cytometry of the intact TU-BcX-2 K1 tumor pieces that were passaged in mice. TU-BcX-2 K1 tumor has a large population of CSCs and in the circulating cells isolated from the peripheral blood of the mice at sacrifice. Both human (HLA^+^) and mouse (HLA^−^) cells within the circulating cells had high CSC populations (Fig. 2c). Tumor pieces were then plated in low-attachment culture conditions in the presence of Matrigel™ (also known as primary spheres) for examination of the CSC population. TU-BcX-2 K1 primary spheres grown under non-adherent conditions, contain cells that exhibit a cancer stem cell-like phenotype. These cells can be characterized by a CD44^+^CD24^low^ flow cytometry profile, though other markers have been used as well, e.g. CD90 or ALDH1 isoforms [25, 26] (Additional file 3: Figure S3A). We use z-stack imaging to show a representative sphere budding off the tumor explant (Additional file 3: Figure S3B).

Next, we transitioned to mouse models to demonstrate that our TU-BcX-2 K1-derived cells were tumorigenic. TU-BcX-2 K1 adherent cells formed tumors after inoculation into a SCID/beige mouse, although there was a notable longer latency for tumors to form compared to implanted TU-BcX-2 K1 tumors (Additional file 4: Figure S4A). At necropsy, the tumor, lungs and liver were fixed and processed for histological analyses. H & E staining of the resulting cell line-derived tumor revealed similar cytology as compared to the original TU-BcX-2 K1 tumors that were passaged in mice (specifically passage 2 and passage 5). However, we did observe a loss of tumor stroma in our cell line-derived tumor compared to the original PDX implanted tumors (Additional file 4: Figure S4B), indicating that implantation of intact tumors maintained the stromal composition of tumors in mouse models. TU-BcX-2 K1-derived cells are capable of forming tumors in vivo*,* although the resulting tumors do not recapitulate the fibrotic architecture of the original PDX tumor from which the cells were derived. At necropsy, we harvested the lungs and liver from the mouse injected with TU-BcX-2 K1-derived cells to evaluate metastatic formation capabilities of the cell line. Mice injected with TU-BCx-2 K1 cells resulted in larger metastatic lesions in both the lungs and liver compared to TU-BCx-2 K1 original implants passaged in mice (Additional file 4: Figure S4C).

Matrigel™ is secreted by murine tumors and is a mixture of extracellular matrix (ECM) including laminin-111, collagen IV, heparan sulfate proteoglycan, growth factors, and other components [27]. Matrigel is consistently used in xenograft experiments in cancer research because, when combined with cancer cells, it increases both the ‘take’ and growth of tumors in mice [28]. However, the resulting tumors formed do not accurately recapitulate the unique stromal architecture or complex cell-cell contacts found in patient tumors [28]. To demonstrate this, we treated TU-BCx-2 K1 explants with collagenase to isolate the cellular population(s) from the stroma. Then, we inoculated SCID/Beige mice with collagenase-digested tumors mixed with Matrigel™. The resulting tumor had a similar gross appearance to the original intact TU-BCx-2 K1 tumor pieces (Additional file 5: Figure S5A). We observed a loss of histological architecture, specifically fibrotic areas, in the H & E stained tumor sections of the collagenase-treated tumors compared to the original TU-BcX-2 K1 tumor, while cell histology was similar (Additional file 5: Figure S5B). Then, to observe if the sphere-forming capabilities of the tumor was affected by disruption of the ECM, we plated the digested tumor in 3D culture conditions to form primary spheres. We show previously the original TU-BcX-2 K1 tumor can form large primary spheres, and the collagenase-digested tumors maintained the same sphere-forming capabilities when plated in 3D culture conditions. Finally, we compared gene expressions of the collagenase-digested tumor that formed after implantation in mice, to original TU-BcX-2 K1 tumors passaged in mice, as well as to the tumor that formed after TU-BcX-2 K1 cells were injected into mice. Because the resulting tumors in each of the three groups exhibited distinct characteristics, we wanted to observe similarities in endogenous gene expressions. Amongst the three groups, we found differences in endogenous expressions of both the epithelial gene *CDH1* as well as the mesenchymal genes *VIM* and *CDH2* (Additional file 6: Figure S6A). Expression of all three genes were suppressed in both the collagenase-digested tumor as well as the cell line-generated tumor compared to the original PDX tumor implants. These findings that collagenase-treatment alters cell characteristics were observed in another PDX model, TU-BCx-2O0 (Additional file 6: Figure S6B). Together, these data demonstrate that digestion of stromal components with collagenase results in altered gene expressions and changes in characteristics of TU-BcX-2 K1 and implicates the importance of the ECM and the stromal architecture in tumorigenesis.

### Oncology drugs confer sensitivity and resistance to TU-BcX-2 K1-derived adherent cells

**Fig. 3:**
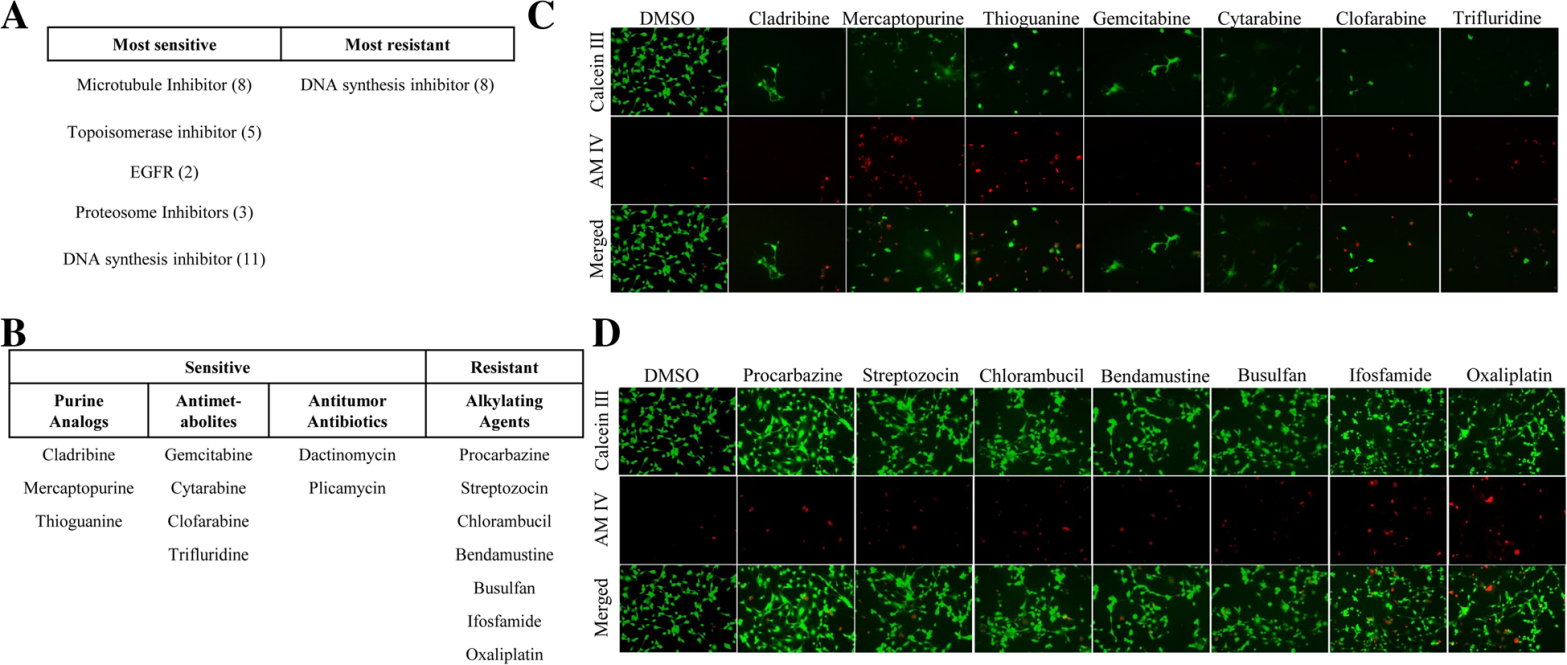
TU-BcX-2 K1 adherent cells’ response to NCI drug panel. TU-BcX-2 K1 cells were treated for 72 h with the NCI oncology drug set (1 μM) or DMSO controls. Cells were harvested, stained with Calcein-AM and EthD III and fluorescence used to visualize the live and dead cells. Green = Calcein-AM (live cells), Red = EthD III (dead cells). **a** Initial observations of imaged TU-BcX-2 K1 cells after treatment showed that TU-BcX-2 K1 cells were most sensitive to compounds targeting microtubules, topoisomerases, proteasomes, EGFR and DNA synthesis inhibitors. TU-BcX-2 K1 cells were most resistant to a subset of DNA synthesis inhibitors. Drugs that were considered to have the ‘most sensitive’ effect on TU-BcX-2 K1 cells resulted in either no cells remaining after treatment, or all dead stained cells. Drugs that were considered to have the ‘most resistant’ effect on TU-BcX-2 K1 cells resulted in all viable (green) cells after treatment. **b** Among the most effective DNA synthesis inhibitors, TU-BcX-2 K1 cells were sensitive to the specific drug classes: purine analogs, antimetabolites, anticancer antibiotics. TU-BcX-2 K1 cells were most resistant to alkylating agents within the larger classification of DNA synthesis-targeting compounds. **c** Representative images of purine analogs and antimetabolites to which TU-BcX-2 K1 was most sensitive. **d** Representative images of alkylating agents that were not effective in TU-BcX-2 K1 cells. Images were captured using fluorescence microscopy and are shown at 100X magnification

We used TU-BcX-2 K1-derived cells to evaluate the effects of specific chemotherapy drugs in different cell culture conditions. For these experiments we employed a live/dead fluorescence stain and a set of clinically approved oncology drugs provided by the National Cancer Institute (NCI). For our initial experiments, we evaluated chemosensitivity of TU-BCX-2 K1 cells plated in adherent, 2D cell culture conditions. First, we identified the systemic chemotherapy agents that were most cytotoxic to TU-BcX-2 K1-derived cells. Our screen was performed in a blind approach, in that drugs that correlated to individual wells were unknown until after images were captured. We found patterns in inhibitors that had the most cytotoxic effects in 2D culture. In the initial analysis, drugs that were considered to have the ‘most sensitive’ effect on TU-BcX-2 K1 cells either 1) resulted in zero cells remaining (live or dead) in the wells or 2) resulted in all stained cells to be dead (red) after treatment and staining. Drugs that were considered to have the ‘most resistant’ effect on TU-BcX-2 K1 cells resulted in all viable (green) cells after treatment and staining. TU-BcX-2 K1 cells were most sensitive to DNA synthesis inhibitors, microtubule inhibitors, and topoisomerase inhibitors (Fig. 3a). Of the DNA synthesis inhibitors among the most effective chemotherapies, we observed patterns in inhibitors of similar classes. This included the antitumor antibiotics (dactinomycin, plicamycin), purine analogs (cladribine, mercaptopurine, thioguanine) and antimetabolites (gemcitabine, cytarabine, clofarabine, trifluridine) (Fig. 3b-c). The most sensitive microtubule inhibitors were plant alkaloids (vincrisitine, vinblastine, vinorelbine, docetaxel, cabazitaxel, paclitaxel) (Additional file 7: Figure S7). Concordantly, we identified the systemic chemotherapies to which TU-BcX-2 K1 cells were most resistant. DNA synthesis inhibitors were the only class of inhibitors that were resistant. Interestingly, the DNA synthesis inhibitors that were not cytotoxic to TU-BcX-2 K1 cells were alkylating agents (procarbazine, streptozocin, chlorambucil, bendamustine, busulfan, ifosfamide, oxaliplatin, mechlorethamine) (Fig. 3d). TU-BcX-2 K1 cells were also resistant to treatment with the pharmacologically similar alkylating agents cyclophosphamide, carmustine, uramustine and lomustine (Additional file 8: Figure S8).

**Fig. 4:**
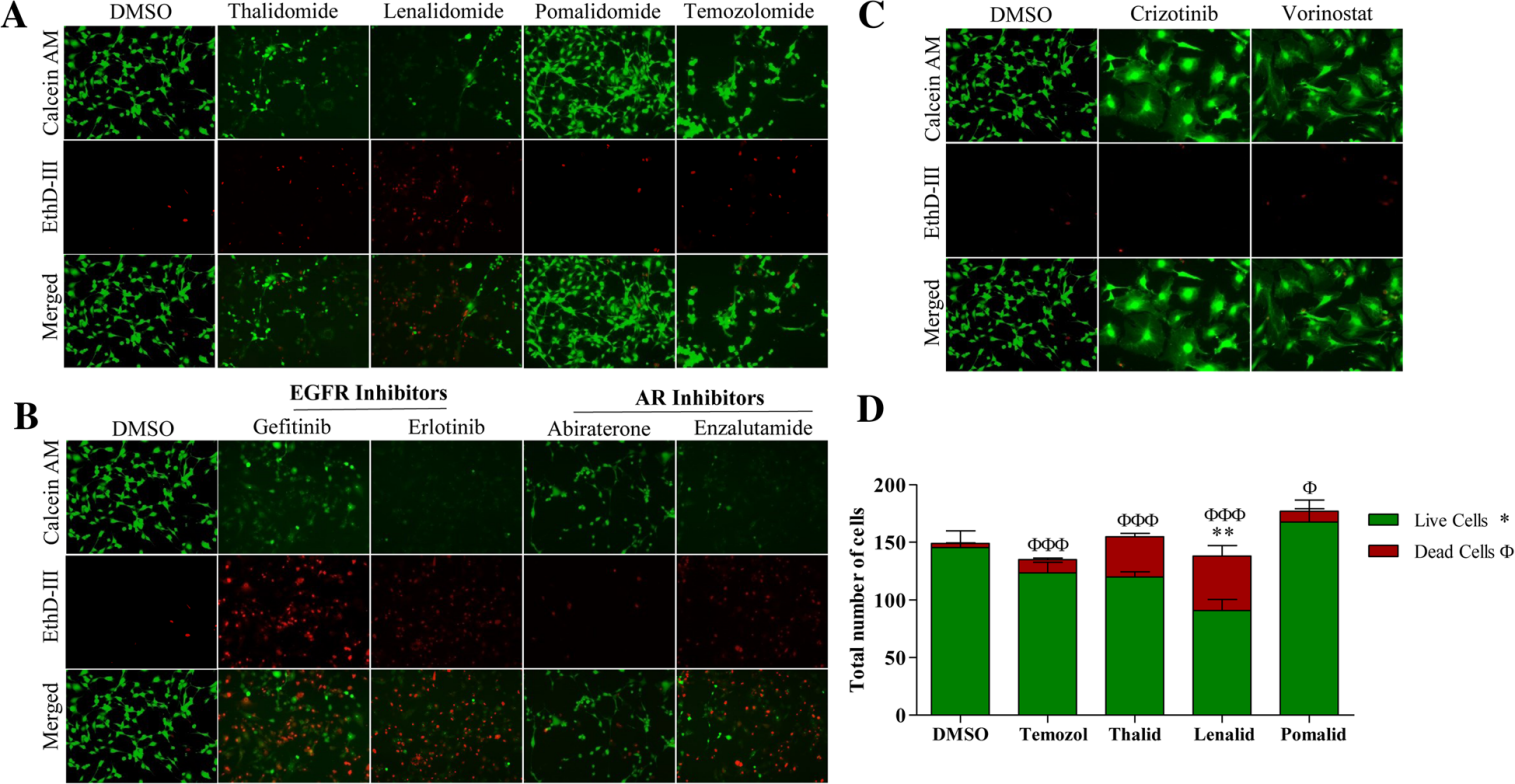
Chemosensitivity profiles of TU-BcX-2 K1 amongst specific drug classes and targeted small molecule inhibitors. TU-BcX-2 K1 cells were treated for 72 h with the NCI oncology drug set (1 μM) or DMSO controls. Cells were harvested, stained with Calcein-AM and EthD III and fluorescence used to visualize the live and dead cells. Green = Calcein-AM (live cells), Red = EthD III (dead cells). **a** Representative images of stained TU-BcX-2 K1 cells showing varying therapeutic responses of TU-BcX-2 K1 cells to specific drugs within the cereblon inhibitors drug class. **b** Small molecule targeted inhibitors within the same drug class had different efficacy on TU-BcX-2 K1 cells. **c** Small molecule kinase inhibitors from the NCI oncology screen that reverse the mesenchymal morphology of TU-BcX-2 K1 cells included cirzotinib and vorinostat. All images were captured with fluorescence microscopy at 100X magnification. **d** Quantification of live and dead cells from the cereblon inhibitors drug class in panel A. Data is represented as total number of live compared to total number of dead cells. Significance was obtained by comparing numbers of live and dead cells in the treatment groups to respective DMSO controls. The symbol ‘^*^’ represents significance for live cell comparisons, and ‘^ф^’ shows significance for dead cell comparisons. * *p* < 0.05, ** *p* < 0.01, *** *p* < 0.001

These initial observations led us to further investigate drug classes to which TU-BcX-2 K1 is most responsive. We observed differences in chemosensitivity amongst specific inhibitors within various drug classes. For example, within the cereblon inhibitors in the NCI panel, TU-BcX-2 K1 cells were most resistant to pomalidomide, and most sensitive to thalidomide and lenalidomide (Fig. 4a). These observations of different cytotoxic responses amongst pharmacologically similar compounds were consistent with small molecule targeted inhibitors as well. Although TU-BcX-2 K1 cells were sensitive to both gefitinib and erlotinib EGFR inhibitors, more viable cells remained after gefinitib treatment. Similarly, we observed that between the androgen receptor inhibitors abiraterone and enzalutamide, more viable cells remained after abiraterone treatment (Fig. 4b). However, this may be due to off-target effects since abiraterone is a steroid-based inhibitor and is activated after metabolism through cytochrome-P450s. These data demonstrate different responses to inhibitors within the same drug class. Next, we found two drugs in our screen that dramatically affected the morphology of TU-BcX-2 K1 cells without reducing cell viability. The two compounds were crizotinib, a small molecule inhibitor targeting ALK, ROS and c-MET, and vorinostat, a histone deacetylase inhibitor (Fig. 4c). Although cell morphology was affected, cell viability was not suppressed. The observations of differences in cell response to compounds within the same drug class with both systemic chemotherapies and targeted small molecule inhibitors was confirmed after the relative numbers of live and dead cells were quantified (Fig. 4d).

### Differences in chemosensitivity observed in TU-BcX-2 K1-derived cells plated in 2D compared to 3D cell culture conditions

**Fig. 5:**
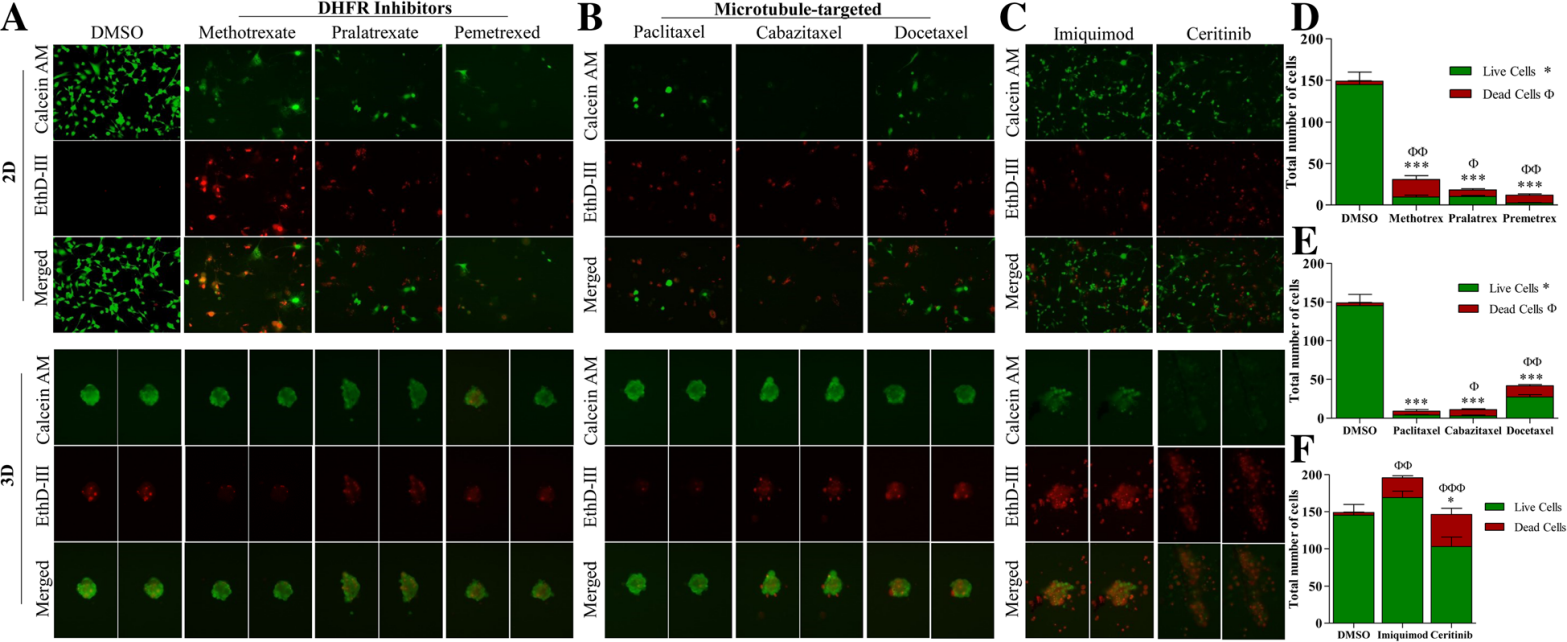
Compounds identified from screen that had different chemosensitivity profiles in 2D and 3D culture conditions. TU-BcX-2 K1 cells were plated in 2D and 3D conditions and treated for 72 h with the NCI oncology drug set (1 μM) or DMSO controls. Cells were harvested, stained with Calcein-AM and EthD III and fluorescence used to visualize the live and dead cells. Green = Calcein-AM (live cells), Red = EthD III (dead cells). **a** Therapies targeting dihydrofolate reductase (**a**) and microtubules (**b**) that were efficacious in adherent plated TU-BcX-2 K1 cells but ineffective in 3D conditions. **c** Small molecule targeted inhibitors that had limited efficacy in 2D/adherent conditions but were efficacious in three-dimensional cell conditions. (D-F) Quantification of live and dead cells from the selected inhibitors in panels (**a-c**). Data is represented as total number of live compared to total number of dead cells. Significance was obtained by comparing numbers of live and dead cells in the treatment groups to respective DMSO controls. The symbol ‘^*^’ represents significance for live cell comparisons, and ‘^ф^’ shows significance for dead cell comparisons. * *p* < 0.05, ** *p* < 0.01, *** *p* < 0.001. All data was obtained after adherent TU-BcX-2 K1 cells were treated and subsequently staining with Calcein-AM to highlight live cells (green) or EthD-III to highlight dead cells (red). Images were captured with fluorescence microscopy and are shown at 100X magnification

Previous studies have reported that cells plated in 3D culture/sphere conditions recapitulate molecular characteristics that are more reflective of the original tumor, compared to cell plated in adherent/2D culture conditions. Next, we compared therapeutic treatments in 2D and 3D culture conditions to evaluate this observation. Utilization of live/dead staining techniques with the NCI panel also led us to observe dramatic differences in response to drugs in select compounds when cells were plated in 2D cell culture compared to 3D cell culture. These observations were consistent in both systemic chemotherapies and small molecule targeted therapies. TU-BcX-2 K1 cells were sensitive to dihydrofolate reductase (DHFR) inhibitors (methotrexate, pralatrexate, pemetrexed) in 2D conditions. Interestingly, when plated in 3D conditions, TU-BcX-2 K1 cells were more resistant to the DHFR inhibitors at the same dose (Fig. 5a, d). The spheres remained intact after treatment, and cells within the spheres were viable. Similar findings were observed with microtubule-targeting agents (paclitaxel, cabazitaxel, docetaxel): spheres in 3D culture conditions were more resistant to the inhibitors than TU-BcX-2 K1 cells plated in 2D conditions. However, we found the responses in the spheres to be dependent on specific inhibitors. Docetaxel treatment resulted in the presence of more dead cells compared to paclitaxel, where the cells in the spheres remained viable after treatment (Fig. 5b, e). We confirmed our observations were not dose-dependent after treating 2D and 3D plated TU-BcX-2 K1 cells with varying doses of paclitaxel (Taxol). While adherent/2D TU-BcX-2 K1 cells were very responsive to 10 μM, 1 μM and 100 nM doses of Taxol (Additional file 9: Figure S9A), spheres were resistant to treatment at these doses, with only slight cytotoxicity occurring at the 10 μM treatment (Additional file 9: Figure S9B). Another major observation in comparing chemosensitivity in 2D and 3D culture was that although some targeted small molecule inhibitors only had a limited effect on TU-BcX-2 K1 cells in 2D culture conditions, these compounds were cytotoxic to the 3D spheres (Fig. 5c, f). These included imiquimod, a toll-like receptor 7 agonist, and ceritinib, an ALK tyrosine kinase inhibitor.

**Fig. 6:**
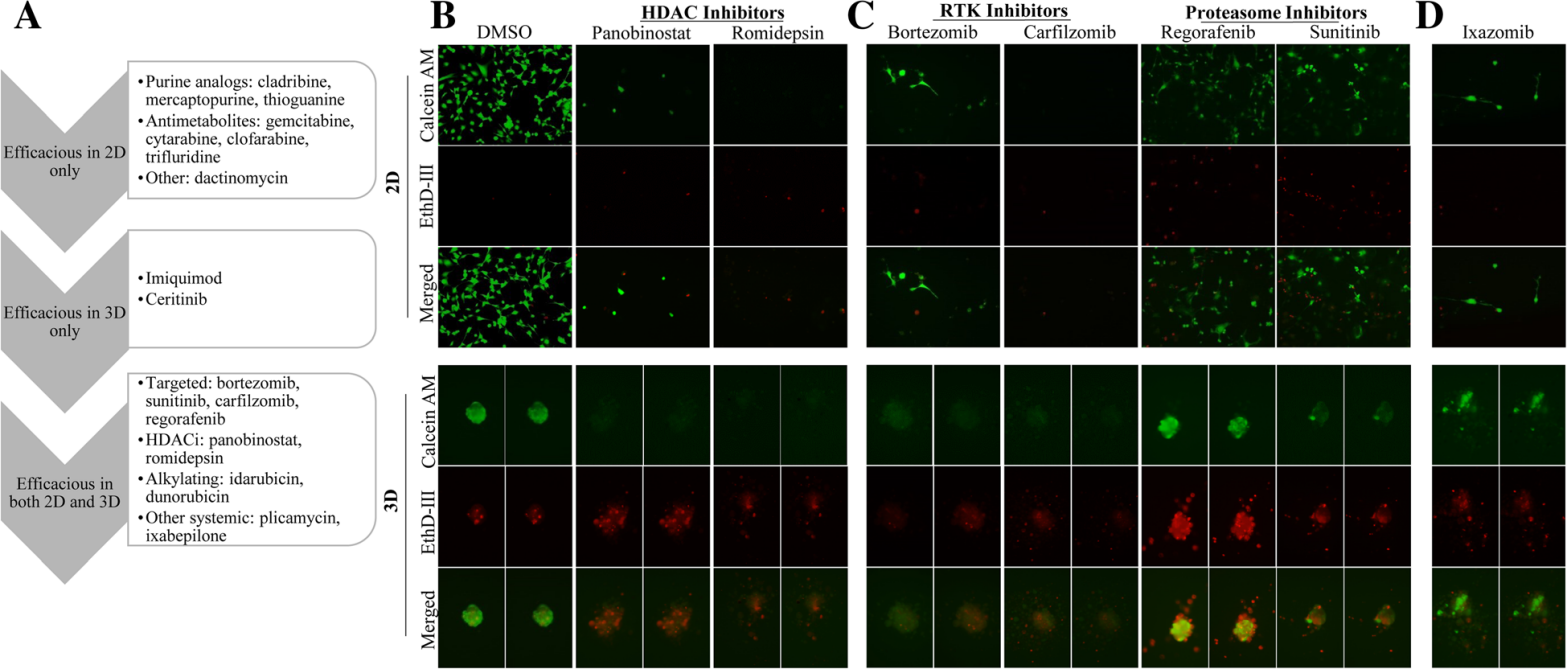
Cytotoxic responses of oncology drugs in TU-BcX-2 K1 cells plated in 2D differ compared to 3D culture conditions. TU-BcX-2 K1 cells were treated for 72 h with the NCI oncology drug set (1 μM) or DMSO controls. Cells were harvested, stained with Calcein-AM and EthD III and fluorescence used to visualize the live and dead cells. Green = Calcein-AM (live cells), Red = EthD III (dead cells). **a** Schematic showing oncology drugs that were effective in 2D culture, 3D culture and drugs that were effective in both 2D and 3D conditions. **b** The histone deacytelase inhibitors panobinostat and romidepsin were most cytotoxic to TU-BcX-2 K1 cells in both 2D and 3D culture. **c** Small molecule targeted inhibitors that were cytotoxic to TU-BcX-2 K1 in both 2D and 3D culture. The proteasome inhibitors bortezomib and carfilzomib were cytotoxic to both 2D and 3D-plated cells; TU-BcX-2 K1 cells were resistant to receptor tyrosine kinase inhibitors regorafenib and sunitinib in both 2D and 3D culture conditions. **d** The small molecule targeted inhibitor ixazomib was cytotoxic in 2D-plated TU-BcX-2 K1 cells but did not affect TU-BcX-2 K1 spheres in 3D culture. All data was obtained after adherent TU-BcX-2 K1 cells were treated and subsequently staining with Calcein-AM to highlight live cells (green) or EthD-III to highlight dead cells (red). Cells were treated at 1 μM using the NCI oncology panel. Images were captured at 100X magnification

Our observation that PDX-derived cell plated in 2D culture conditions had different chemosensitivity compared to cells plated in 3D conditions led us to further examine this effect. When we compared treatment of TU-BcX-2 K1 cells in 2D and 3D culture conditions, we identified oncology drugs that were cytotoxic and were effective in 2D culture alone, in 3D culture alone, and drugs that were cytotoxic in both 2D and 3D culture conditions (Fig. 6a). The HDAC inhibitors panobinostat and romidepsin were cytotoxic in both 2D and 3D culture conditions (Fig. 6b). Interestingly, we also found different responses in TU-BcX-2 K1 cells of targeted inhibitors that have similar targets in 2D compared to 3D culture conditions. Carfilzomib and bortezomib are selective proteasome inhibitors, and sunitinib and regorafenib have activity against multiple receptor tyrosine kinases (RTKs), including PDGFRs and VEGFRs. TU-BcX-2 K1 cells in both 2D and 3D culture conditions were sensitive to proteasome inhibitors (Fig. 6c). With respect to the RTK inhibitors, TU-BcX-2 K1 cells in both 2D and 3D conditions were sensitive to treatment with sunitinib, but TU-BcX-2 K1 cells were more resistant to regorafenib in 3D conditions, with more viable cells remaining (Fig. 6c). Because the RTK inhibitors have different anti-kinase activities, these data suggest TU-BcX-2 K1 cells are less responsive to one of the multiple kinase targets of regorafenib. Finally, we previously identified systemic chemotherapeutics that were cytotoxic to TU-BcX-2 K1 cells in 2D, but not in 3D conditions. In follow-up observations we found therapies that were sensitive in 2D culture and resistant to 3D. Ixazomib was cytotoxic in 2D but spared 3D spheres (Fig. 6d).

### TU-BcX-2 K1 cells respond to HDAC inhibition

In our screen approach using the NCI clinically approved oncology drug set, we observed TU-BcX-2 K1 cells are responsive to HDAC inhibition in both 2D and 3D culture conditions but were resistant to paclitaxel treatment in 3D conditions. We interrogated this observation using primary sphere culture of TU-BcX-2 K1 cells embedded in Matrigel™, in which tumor pieces are plated in 3D conditions and spheres grow from the explants. Previously in this manuscript we showed that TU-BcX-2 K1 primary mammospheres, or spheres derived from tumor explants under non-adherent conditions, contain cells that exhibit a cancer stem cell-like phenotype.

We used romidepsin, and HDAC inhibitor, to interrogate cytotoxic effects of HDAC inhibition compared to paclitaxel treatment in 3D conditions. We pre-treated TU-BcX-2 K1 primary spheres with DMSO control, romidepsin and paclitaxel (Taxol), and embedded the spheres in Matrigel™. Immunofluorescence of CSC markers revealed that while romidepsin suppressed the CSC population in the spheres, treatment with taxol increased the CSC population, specifically increasing the CD44^+^ cells (Additional file 10: Figure S10). These observations are consistent with our findings in the initial drug screen.

**Fig. 7:**
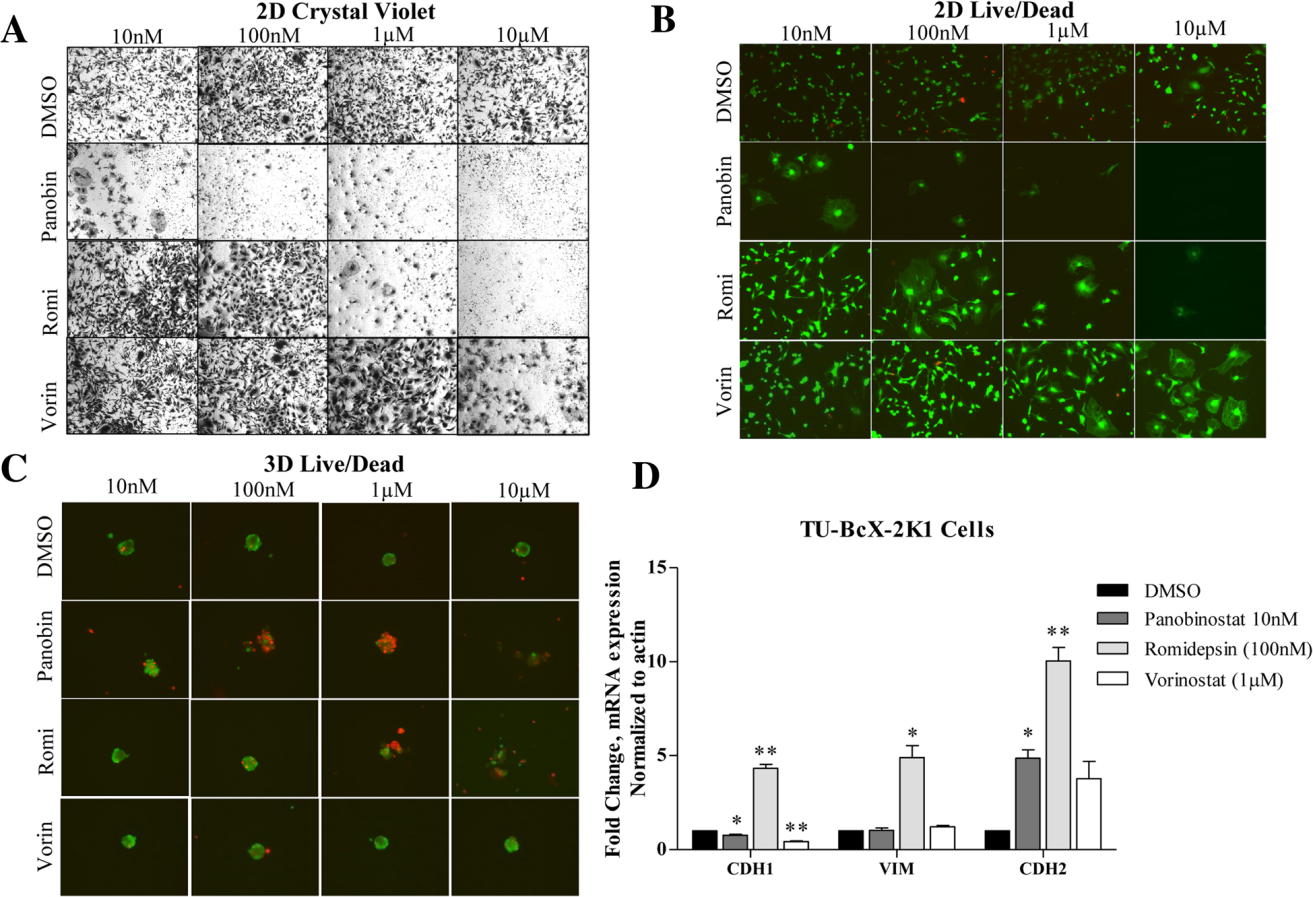
Differential response of TU-BcX-2 K1 to HDAC inhibitors in 2D and 3D culture conditions. TU-BcX-2 K1 cells treated with the HDAC inhibitors panobinostat (panobin), romidepsin (romi) and vorinostat (vorin). Cell viability and morphology were visualized using **a** crystal violet staining in 2D conditions, **b** live/dead fluorescence staining in 2D conditions and **c** live/dead staining in 3D conditions. In the live/dead staining, live cells are stained with Calcein-AM (green), dead cells stained with EthD-III (red). Images were captured at 100X magnification. **d** Morphology-related gene changes after adherent TU-BcX-2 K1 cells treated with panobinostat, romidepsin and vorinostat. qRT-PCR was used to evaluate gene expression and results were normalized to β-actin and DMSO vehicle controls. Significance is represented as follows: * *p* < 0.05, ** *p* < 0.01, *** *p* < 0.001. Error bars represent S.E.M. *N* = 3

In the screen, we observed a differential response of TU-BcX-2 K1 cells to the HDAC inhibitors panobinostat, romidepsin and vorinostat. In 2D culture conditions at the same dose (1 μM), panobinostat and romidepsin were cytotoxic to TU-BcX-2 K1 cells, while vorinostat was less cytotoxic but altered the cell phenotype dramatically. To evaluate if these observations were dose-dependent or drug-dependent, we repeated the live/dead stain in addition to crystal violet staining at varying doses. We confirmed that vorinostat, or SAHA, was least effective in TU-BcX-2 K1 cells with an IC50 of 1 μM and panobinostat was most effective, with an IC50 of 10 nM. All three HDAC inhibitors reversed the mesenchymal morphology of the cells at their respective optimal doses, and this was a non-cytotoxic effect (Fig. 7a-b). In 3D culture conditions we observed a similar pattern in cytotoxicity: panobinostat was the most cytotoxic HDAC inhibitor compared to vorinostat at romidepsin, with dramatic effects occurring at 100 nM (Fig. 7c). Vorinostat was the least effective HDAC inhibitor, with minimal effects on cytotoxicity of TU-BcX-2 K1-derived spheres at all doses. Cytotoxicity due to romidepsin treatment started at the 1 μM dose.

Next, we examined if the observed cell morphology effects translated to alteration of morphology-associated gene signatures. For these experiments, we used the epithelial marker *CDH1*, of which higher mRNA expression is associated with the epithelial cell phenotype, and the mesenchymal markers *VIM* and *CDH2*, which are associated with mesenchymal cell phenotypes. In 2D conditions in follow-up qRT-PCR analyses, treatment of TU-BcX-2 K1 cells with panobinostat (10 nM) suppressed *CDH1* and significantly increased *CDH2* mRNA expressions. Romidepsin (100 nM) increased *CDH1, VIM* and *CDH2* mRNA expressions. Vorinistat suppressed *CDH1* and increased *CDH2* expression, although not significantly. These data indicate different epithelial and mesenchymal gene expression responses of TU-BcX-4IC cells to the different HDAC inhibitors. (Fig. 7d). In contrast, panobinostat-treated TU-BcX-2 K1 tumor explants increased *CDH1* and suppressed *VIM* and *CDH2* mRNA expression, potentially indicating trans-differentiation to a more luminal-like phenotype. Although romidepsin increased *CDH1* expression, it also increased *VIM* and *CDH2* expression in TU-BcX-2 K1 explants in a similar pattern to treated PDX-derived cells (Additional file 11: Figure S11). These data show that gene analyses of in vitro cell line treatments with the pan-HDAC inhibitor was not concordant with treatments of tumor explants ex vivo. However, romidepsin treatment both increased the epithelial marker *CDH1* expression in TU-BcX-2 K1 cells, and the gene expression patterns were similar in treatment of PDX-derived cells and tumor pieces ex vivo.

## Discussion

Targeted therapy agents for triple negative breast cancer are difficult to develop due to the lack of broadly expressed targetable receptors. First-line treatment regimens for triple negative breast cancer cases use cytotoxic chemotherapies; there are no clinically-approved small molecule targeted therapies for TNBC. Discovery of novel targeted therapies is encumbered by inadequate preclinical models that do not accurately translate in vitro findings into the clinic. Better preclinical predictive models, including spheroids, organoids and PDX, are urgently needed. Here, we describe a new translational model and a high-content technique to evaluate chemosensitivity of patient-derived TNBC cells in the laboratory setting. Patient-derived xenografts (PDXs) are better preclinical model than immortalized cell line models because they recapitulate unique characteristics that are present in the original patient tumor. Although triple negative breast cancer has higher mortality rates in African-American women, patient-derived xenograft tumors from patients with African ancestry are underrepresented [9]. In this study, we first characterized TU-BCx-2 K1, a new TNBC PDX model established from an African-American patient in our laboratory. After implantation in immunocompromised mice, we found that TU-BCx-2 K1 minimally metastasized to the lungs and livers. TU-BcX-2 K1 had a successful tumor take in mice with every passage and formed spheres when plated in low-suspension/3D culture conditions.

Acquisition of drug resistance results in fatal outcomes for patients with recurrent tumors. We utilized cells derived from our TU-BcX-2 K1 model to compare chemosensitivity in cells plated in 2D adherent and 3D/sphere culture conditions. In these experiments, we use the NCI approved oncology drug set, which contains over 125 commonly used and clinically approved oncology drugs. Many groups use techniques such as crystal violet staining, MTT assays, luciferase or other assays to evaluate chemoresponse and cytotoxicity. The cytotoxicity-evaluating techniques (MTT, luciferase, SRB assays) are time-intensive, require significant skill and can produce fluctuating results with different culture conditions [29]. Crystal violet staining does not facilitate more in-depth observations regarding the response to therapies, such as the percentage of live compared to dead cells. We found that using a live/dead staining kit facilitates identification of inhibitor classes or specific inhibitors that are most or least effective in TU-BcX-2 K1 cells. Calcein-AM penetrates the cell membrane of living cells and hydrolyzes cellular esterases to become cell membrane-impermeable, green fluorescent Calcein. EthD-III only passes through the membranes of dead cells and then intercalates with nuclear DNA, emitting red light. This live/dead stain is easy to use, provides immediate results, and can be analyzed using a variety of techniques (fluorescence microscopy, spectrophotometry, flow cytometry) to provide descriptive information about specific therapeutic responses. However, it is important to note that although Calcein and Ethidium homodimer staining can be used to assess the cytotoxicity of tested compounds, it is unable to detect any cytostatic effect.

Using this method, we found that TU-BcX-2 K1 cells were most sensitive to microtubule inhibitors, topoisomerase inhibitors, and DNA synthesis inhibitors. In contrast, TU-BcX-2 K1 cells were also resistant to DNA synthesis inhibitors. When we looked at specific classes of DNA synthesis inhibitors, we observed that TU-BcX-2 K1 was sensitive to purine analogs, antimetabolites and antitumor antibiotics, and resistant to cereblon inhibitors. This approach not only highlights if a drug class was effective in individual patient’s tumor cells but allows us to quantify the effectiveness of specific chemotherapies within drug classes. For example, within the immunomodulatory antineoplastic drug class, we found that TU-BcX-2 K1 cells were resistant to pomalidomide treatment, but more responsive to thalidomide and lenalidomide. Lenalidomide treatment resulted in more EthD-positive, or dead, cells. Immunomodulatory drugs have diverse targets in the immune system and tumor microenvironment, and specific mechanisms remain unknown. Pomalidomide is the newest and most selective member of the class [30, 31], and because TU-BcX-2 K1 cells were more resistant to pomalidomide, this suggests TU-BcX-2 K1 cells were inherently resistant to one of the selective targets of pomalidomide. Altered cell morphology is another aspect of chemosensitivity that can be evaluated using the live/dead stain. Only two drugs in the NCI panel had dramatic effects on cell morphology in TU-BcX-2 K1 cells at the 1 μM dose without affecting viability: crizotinib and vorinostat. Both compounds are targeted small molecule inhibitors, with crizotinib targeting ALK, ROS and c-MET, and vorinostat targeting histone deacetylase (HDAC) enzymes (class I, II, IV). Our data support a role of ALK, ROS, c-MET and/or HDAC deacetylase enzymes in controlling cytoskeletal rearrangement and cell morphology. Future studies are required to parse out which kinase(s) are responsible for the morphology observations.

Most cytotoxic and targeted agents in preclinical studies use 2D adherent cell culture conditions to evaluate response given the speed of use and their scalability. Ex vivo models that do not require a passage in mice, such as tumor-derived spheroids or organoids, can be used to rapidly screen classes of drugs for individual patients [32]. PDX-derived cells grown in low-attachment, or 3D, conditions exhibit behaviors and phenotypes that more accurately represent cells in physiologic settings. When we compared chemosensitivity to TU-BcX-2 K1 cells in 2D and 3D culture, we observed distinct differences. Our observations that targeted small molecule inhibitors (imiquimod and ceritinib) that had a limited effect in 2D culture but had dramatic responses in 3D culture has significant implications for targeted therapeutic discovery. Conversely, we identified targeted agents that were cytotoxic to TU-BcX-2 K1 cells plated in 2D culture but did not affect 3D-plated spheres, for example ixazomib. We show that PDX-derived cells grown in two different conditions have profoundly different responses to oncology drugs.

We also report observations that support the hypothesis that HDAC inhibitors reverse the mesenchymal phenotype in cancer cells, independent of cytotoxicity. The effect of HDAC inhibitors on the epithelial-to-mesenchymal transition (EMT), mechanistically through upregulation of E-cadherin, varies in different cancer types [33, 34]*.* In breast cancer, HDAC inhibitors have been found to reverse EMT [35, 36], and we have shown this is confirmed in TNBC cells specifically [37, 38]. Here we show that all the HDAC inhibitors in the NCI oncology drug panel (panobinostat, romidepsin and vorinostat) reverse the mesenchymal cell morphology in a dose-dependent manner in our TNBC PDX model. Interestingly, these results were not translated changes in mesenchymal-related gene expressions of TU-BcX-2 K1 adherent cells. Only romidepsin increased *CDH1* expression, and all three HDAC inhibitors had minimal effects on *VIM* and *CDH2*. However, when intact TU-BcX-2 K1 explants were treated, we observed different changes in EMT-related genes in the panobinostat group, specifically a reversal of the EMT phenotype (increase in *CDH1*, suppression of *VIM* and *CDH2*). These data show that treatment of adherent PDX-derived cells in adherent, or 2D culture conditions for some anticancer agents tested in vitro does not translate to treating the matched intact patient-derived tumors.

Importantly, we show that our observations from the adherent and suspension treatment screen can be translated into the treatment of PDX tumor-derived spheres. For example, in the live/dead screen we found paclitaxel to have minimal effect on the viability of spheres compared to romidepsin treatment. We observed similar results with treated TU-BcX-2 K1 spheres derived from tumor explants – romidepsin resulted in a suppression of the CSC phenotype (CD44^+^CD24^−^) while Taxol maintained stemness. This is an important observation and contributes to recent findings that resistance to taxanes in TNBC is mediated by enrichment of the tumor-initiating populations [39]. It is also crucial to demonstrate that treating adherent cells in adherent conditions is very different from treatment of tumor explants, which more accurately mimics treating a patient’s tumor. We show that gene expression changes, in response to drug treatment, differ in treatment conditions. These data support the idea that using adherent cells in preclinical therapeutic experiments is not sufficient to translate in vitro findings into clinical applications.

We acknowledge that utilization of the live/dead stain has limitations. Comparing relative quantification values across different drugs is difficult when using this technique because some inhibitors are extremely cytotoxic to the cells, lysing the cells and leaving no remaining cells in the dish to stain after the wash step. Although this demonstrates the cytotoxic effects of the therapy, if one therapy causes 50% of the initial cells to remain in the dish, and another causes 100% of the cells to remain, we cannot directly quantify the number of live and dead cells in these wells. To address this, we compared relative percentages of cells (dead compared to alive). Another limitation of our study is that we treated TU-BcX-2 K1 cells with drugs from the NCI oncology panel at the same concentration. We understand that therapeutic efficacy is dose-dependent for specific oncology drugs, and while we did follow-up dose-response studies with select agents we did not perform follow-up studies for the entire drug set. Also, within the NCI oncology drug set = there are compounds that were cytotoxic to TU-BcX-2 K1 cells, but this effect may have been due to off-target activities. For example, some compounds exert activity through inhibition of cytochrome P450 enzymes (abiraterone), some are pro-drugs which should not be active until metabolized (cyclophosphamide, ifosfamide) and some are immune modulators that exert effects through suppression of cytokines and immune cells (second generation thalidomides). Despite these limitations, our objective was to demonstrate the power of the live/dead stain to evaluate drug response in TU-BcX-2 K1 cells and compare our findings in adherent and suspension culture, and not to pursue specific drugs identified in our screen. While these results provide insight into the role for PDX models in understanding tumor biology and sensitivity to therapeutics, it is important to ultimately correlate these results with patient responses and in vivo systems. PDX, PDX-O and PDX-E models compliment current in vitro cell lines and in vivo cell lines-derived xenografts. However, the impact on our understanding of cancer biology ultimately requires testing our observations in in vivo systems, utilizing the PDX models.

## Conclusions

In summary, we describe the utilization of a preclinical model to evaluate chemosensitivity in the laboratory setting. This method combines the use of PDX models, three-dimensional culture conditions and live/dead staining techniques. We demonstrate the efficacy of this model in TNBC therapeutic discovery research using a new PDX model established by our group at Tulane Cancer Center, which represents a patient with African ancestry which is an under-represented population in TNBC research. This method of evaluating therapeutic efficacy in a preclinical setting can be applied to drug screens in all areas of cancer research and is not limited to breast cancer.

## Additional files

Additional file 1: **Figure S1.** Within the human population (HLA^+^) of TU-BCx-2 K1 explants, there were low populations of both CD31, a marker for angiogenesis, and CD14, a marker for granulocytes. Within mouse populations (HLA^−^), there was a significantly higher population of CD14. Data was obtained using flow cytometry techniques. *N* = 2; *** *p* < 0.005 * *p* < 0.05. (DOCX 29 kb)

Additional file 2: **Figure S2.** Cell lines generated from the TU-BCx-2 K1 tumor were capable of forming spheres in 3D culture conditions. (DOCX 204 kb)

Additional file 3: **Figure S3.** (A) TU-BCx-2 K1 explants were embedded in 40% Matrigel and immunofluorescence was employed to evaluate CD44 (red) and CD24 (green) populations within the spheres. ‘T’ indicates the tumor explant from which the mammospheres budded. Arrows indicate CD44^+^CD24^low^ cells and arrowheads indicate CD44^+^CD24^low^ cells. (B) z-stack imaging of the CD44^+^ immunofluorescence stained explant-derived mammospheres. Red = CD44, Blue = DAPI nuclear stain. (DOCX 302 kb)

Additional file 4: **Figure S4.** Cells derived from TU-BcX-2 K1 are tumorigenic**.** (A) Cells derived from the TU-BcX-2 K1 model formed a tumor when SCID/Beige mice were inoculated with 2.5 × 10^6^ cells. ‘T6’ indicates the cells were derived from a TU-BCX-2 K1 tumor that was passaged six times in mice, and ‘p6’ denotes the cells were passaged six times in cell culture before inoculation. (B) H & E staining of the resulting tumor show a loss of fibrotic areas of the tumor that were present in the original implants. (C) TU-BcX-2 K1 cells metastasize to both the lungs and liver. Organs were harvested, formalin fixed, paraffin-embedded and H & E stained to visualize metastatic lesions. (DOCX 676 kb)

Additional file 5: **Figure S5.** TU-BcX-2 K1 tumor pieces were treated with collagenase type II and re-implanted in the mfp of SCID/Beige mice. (A) The resulting tumor was similar in gross appearance to the natural TU-BcX-2 K1 tumor. (B) H & E staining showed loss of histological architecture and stromal components. (C) Cells derived from the collagenase-treated tumors formed spheres in 3D conditions. (DOCX 532 kb)

Additional file 6: **Figure S6.** Comparison of gene features of collagenase-treated TU-BcX-2 K1 tumor to natural tumor and cell line-derived tumor. Analysis was performed using qRT-PCR. (A) Collagenase treatment resulted in less endogenous expression of CDH1, VIM and CDH2. This reduction was even more prominent in the cell line-derived tumor. (B) Similar results of collagenase-treated tumor compared to natural tumor were observed in another TNBC PDX model established in our laboratory, TU-BcX-2O0. (DOCX 45 kb)

Additional file 7: **Figure S7.** Selected compounds from the NCI oncology panel that were cytotoxic to TU-BcX-2 K1 cells. Vincristine, vinblastine and vinorelbine are all microtubule-targeting agents. (DOCX 47 kb)

Additional file 8: **Figure S8.** Selected compounds from the NCI oncology panel that were resistant to TU-BcX-2 K1 cells. Cyclophosphamide, uramustine, carmustine and lomustine are all alkylating agents. (DOCX 248 kb)

Additional file 9: **Figure S9.** TU-BcX-2 K1 spheres were resistant to treatment with Taxol, independent of the dose used. (A) Adherent culture of TU-BcX-2 K1 cells treated with Taxol at varying doses (10 nM, 100 nM, 1 μM, 10 μM) for 72 h before fixed and stained with Crystal Violet. (B) Sphere culture of TU-BcX-2 K1 cells treated with Taxol at the same varying doses for 72 h before stained with the Live/Dead Kit. Live cells were stained by Calcein-AM (green) and dead cells were stained with EthD-III (red). *N* = 3 for all experiments. (DOCX 182 kb)

Additional file 10: **Figure S10.** TU-BcX-2 K1 primary mammospheres’ response to selected cytotoxic oncology drugs. (A) TU-BCx-2 K1 explants were embedded in 40% Matrigel™ and immunofluorescence was employed to evaluate CD44 (red) and CD24 (green) populations within the spheres. ‘T’ indicates the tumor explant from which the mammospheres budded. (B) z-stack imaging of the explant-derived mammospheres reveals CD44^high^ cells surround the mammosphere. (C) TU-BcX-2 K1 spheres embedded in Matrigel™ were pre-treated with DMSO (0.1%), Taxol (10 nM) and romidepsin (100 nM) for 72 h before immunofluorescence staining with CD44 (red), CD24 (green) and DAPI nuclear stain (blue). Representative images are shown. Images were captured at 50X magnification. (DOCX 381 kb)

Additional file 11: **Figure S11.** Morphology-related gene changes after TU-BcX-2 K1 explants were treated with panobinostat and romidepsin. Genes analyzed were the epithelial gene E-cadherin (CDH1) and the mesenchymal genes vimentin (VIM) and N-cadherin (CDH2). qRT-PCR was used to evaluate gene expression and results were normalized to β-actin and DMSO vehicle controls. Error bars represent S.E.M. *N* = 3. (DOCX 22 kb)

## Abbreviations

2D: Two dimensional
3D: Three dimensional
ACT: Actin
BC: Breast cancer
CDH1: E-cadherin
CDH2: N-cadherin
CSC: Cancer stem cell
DHFR: Dihydrofolate reductase
DMEM: Dulbecco’s Modified Eagle Media
DMSO: Dimethylsulfoxide
DNA: Deoxyribonucleic acid
ECM: Extracellular matrix
EDTA: Ethylenediaminetetraacetic acid
EGFR: Epidermal growth factor receptor
EMT: Epithelial-to-mesenchymal transition
EthD: Ethidium
FBS: Fetal bovine serum
H & E: Hematoxylin and eosin
HDAC: Histone deacetylase inhibitor
HLA: Human leukocyte antigen
IACUC: Institutional Animal Care and Use Committee
IBC: Institutional Biosafety Committee
IRB: Institutional Review Board
NCI: National Cancer Institute
NIH: National Institute of Health
NK: Natural killer
PBS: Phosphate buffered saline
PDX: Patient-derived xenograft
PDX-E: Patient-derived xenograft explant
PDX-O: Patient-derived xenograft organoid
qRT-PCR: Quantitative reverse transcription polymerase chain reaction
SAHA: Suberoylanilide hydroxamic acid (vorinistat)
SCID: Severe combined immunodeficient
SEM: Standard error of mean
TU-BcX-2 K1: Tulane Breast Cancer Xenograft 2 K1
VIM: Vimentin
