# Supplementary material for: Drug resistance profiling of a new triple negative breast cancer patient-derived xenograft model
Source: BMC Cancer. 2019 Mar 7;19:205. doi: 10.1186/s12885-019-5401-2 (PMC6407287; doi:10.1186/s12885-019-5401-2)
Supplement: Supplementary file 1 — Figure S1. Within the human population (HLA+) of TU-BCx-2 K1 explants, there were low populations of both CD31, a marker for angiogenesis, and CD14, a marker for granulocytes. Within mouse populations (HLA−), there was a significantly higher population of CD14. Data was obtained using flow cytometry techniques. N = 2; *** p < 0.005 * p < 0.05. (DOCX 29 kb) [file 12885_2019_5401_MOESM1_ESM.docx]

**Figure S1.** Within the human population (HLA^+^) of TU-BCx-2K1 explants, there were low populations of both CD31, a marker for angiogenesis, and CD14, a marker for granulocytes. Within mouse populations (HLA^-^), there was a significantly higher population of CD14. Data was obtained using flow cytometry techniques. N = 2; *** p < 0.005 * p < 0.05.
