# Supplementary figures and images for: Drug resistance profiling of a new triple negative breast cancer patient-derived xenograft model
Source: BMC Cancer. 2019 Mar 7;19:205. doi: 10.1186/s12885-019-5401-2 (PMC6407287; doi:10.1186/s12885-019-5401-2)

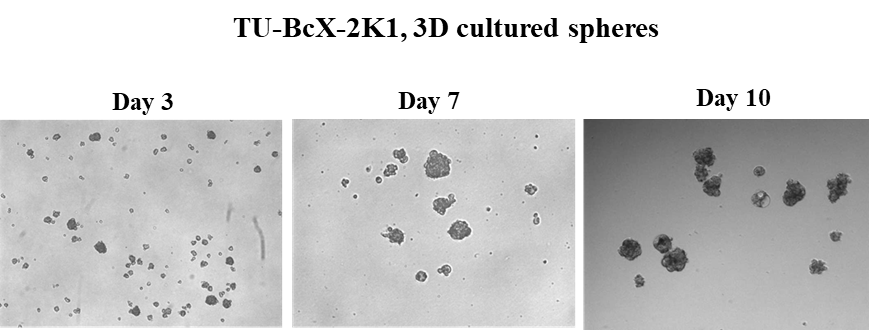


**Figure S2.** Cell lines generated from the TU-BCx-2K1 tumor form spheres in 3D culture conditions.

Supplement: Supplementary file 2 — Figure S2. Cell lines generated from the TU-BCx-2 K1 tumor were capable of forming spheres in 3D culture conditions. (DOCX 204 kb) [file 12885_2019_5401_MOESM2_ESM.docx]
