# Supplementary material for: Drug resistance profiling of a new triple negative breast cancer patient-derived xenograft model
Source: BMC Cancer. 2019 Mar 7;19:205. doi: 10.1186/s12885-019-5401-2 (PMC6407287; doi:10.1186/s12885-019-5401-2)
Supplement: Supplementary file 3 — Figure S3. (A) TU-BCx-2 K1 explants were embedded in 40% Matrigel and immunofluorescence was employed to evaluate CD44 (red) and CD24 (green) populations within the spheres. ‘T’ indicates the tumor explant from which the mammospheres budded. Arrows indicate CD44+CD24low cells and arrowheads indicate CD44+CD24low cells. (B) z-stack imaging of the CD44+ immunofluorescence stained explant-derived mammospheres. Red = CD44, Blue = DAPI nuclear stain. (DOCX 302 kb) [file 12885_2019_5401_MOESM3_ESM.docx]

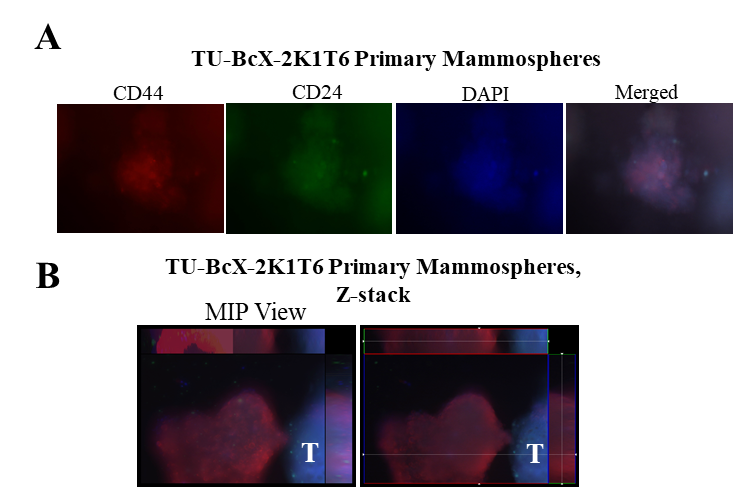


**Figure S3.** (A) TU-BCx-2K1 explants were embedded in 40% Matrigel and immunofluorescence was employed to evaluate CD44 (red) and CD24 (green) populations within the spheres. ‘T’ indicates the tumor explant from which the mammospheres budded. Arrows indicate CD44^+^CD24^low^ cells and arrowheads indicate CD44^+^CD24^low^ cells. (B) z-stack imaging of the CD44^+^ immunofluorescence stained explant-derived mammospheres. Red = CD44, Blue = DAPI nuclear stain.
