# Supplementary material for: Drug resistance profiling of a new triple negative breast cancer patient-derived xenograft model
Source: BMC Cancer. 2019 Mar 7;19:205. doi: 10.1186/s12885-019-5401-2 (PMC6407287; doi:10.1186/s12885-019-5401-2)
Supplement: Supplementary file 4 — Figure S4. Cells derived from TU-BcX-2 K1 are tumorigenic. (A) Cells derived from the TU-BcX-2 K1 model formed a tumor when SCID/Beige mice were inoculated with 2.5 × 10^6^ cells. ‘T6’ indicates the cells were derived from a TU-BCX-2 K1 tumor that was passaged six times in mice, and ‘p6’ denotes the cells were passaged six times in cell culture before inoculation. (B) H & E staining of the resulting tumor show a loss of fibrotic areas of the tumor that were present in the original implants. (C) TU-BcX-2 K1 cells metastasize to both the lungs and liver. Organs were harvested, formalin fixed, paraffin-embedded and H & E stained to visualize metastatic lesions. (DOCX 676 kb) [file 12885_2019_5401_MOESM4_ESM.docx]

**
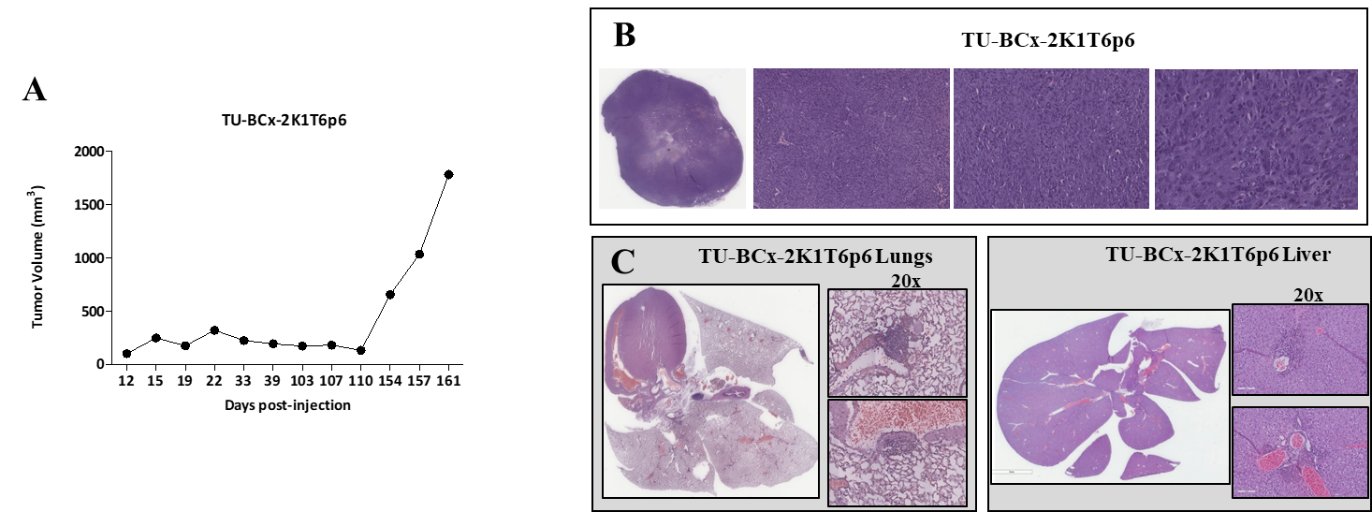
**

**Figure S4. Cells derived from TU-BcX-2K1 are tumorigenic.** (A) Cells derived from the TU-BcX-2K1 model formed a tumor when SCID/Beige mice were inoculated with 2.5 x 10^6^ cells. ‘T6’ indicates the cells were derived from a TU-BCX-2K1 tumor that was passaged six times in mice, and ‘p6’ denotes the cells were passaged six times in cell culture before inoculation. (B) H & E staining of the resulting tumor show a loss of fibrotic areas of the tumor that were present in the original implants. (C) TU-BcX-2K1 cells metastasize to both the lungs and liver. Organs were harvested, formalin fixed, paraffin-embedded and H & E stained to visualize metastatic lesions.
