# Supplementary material for: Drug resistance profiling of a new triple negative breast cancer patient-derived xenograft model
Source: BMC Cancer. 2019 Mar 7;19:205. doi: 10.1186/s12885-019-5401-2 (PMC6407287; doi:10.1186/s12885-019-5401-2)
Supplement: Supplementary file 5 — Figure S5. TU-BcX-2 K1 tumor pieces were treated with collagenase type II and re-implanted in the mfp of SCID/Beige mice. (A) The resulting tumor was similar in gross appearance to the natural TU-BcX-2 K1 tumor. (B) H & E staining showed loss of histological architecture and stromal components. (C) Cells derived from the collagenase-treated tumors formed spheres in 3D conditions. (DOCX 532 kb) [file 12885_2019_5401_MOESM5_ESM.docx]

**
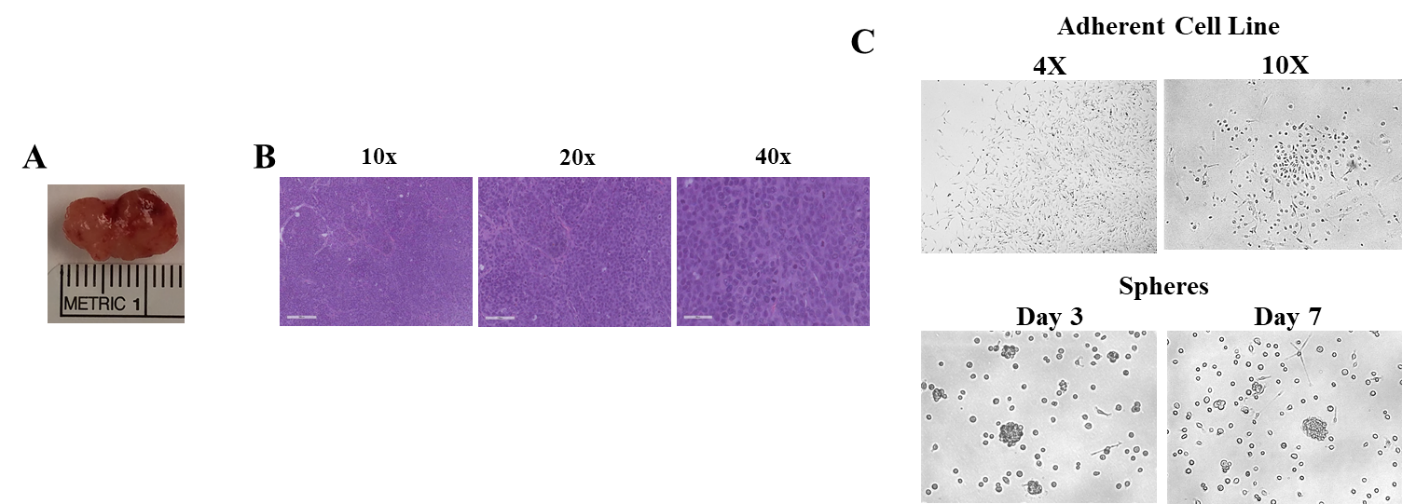
**

**Figure S5.** TU-BcX-2K1 tumor pieces were treated with collagenase type II and re-implanted in the mfp of SCID/Beige mice. (A) The resulting tumor was similar in gross appearance to the natural TU-BcX-2K1 tumor. (B) H & E staining showed loss of histological architecture and stromal components. (C) Cells derived from the collagenase-treated tumors formed spheres in 3D conditions.
