# Supplementary material for: Drug resistance profiling of a new triple negative breast cancer patient-derived xenograft model
Source: BMC Cancer. 2019 Mar 7;19:205. doi: 10.1186/s12885-019-5401-2 (PMC6407287; doi:10.1186/s12885-019-5401-2)
Supplement: Supplementary file 6 — Figure S6. Comparison of gene features of collagenase-treated TU-BcX-2 K1 tumor to natural tumor and cell line-derived tumor. Analysis was performed using qRT-PCR. (A) Collagenase treatment resulted in less endogenous expression of CDH1, VIM and CDH2. This reduction was even more prominent in the cell line-derived tumor. (B) Similar results of collagenase-treated tumor compared to natural tumor were observed in another TNBC PDX model established in our laboratory, TU-BcX-2O0. (DOCX 45 kb) [file 12885_2019_5401_MOESM6_ESM.docx]

**
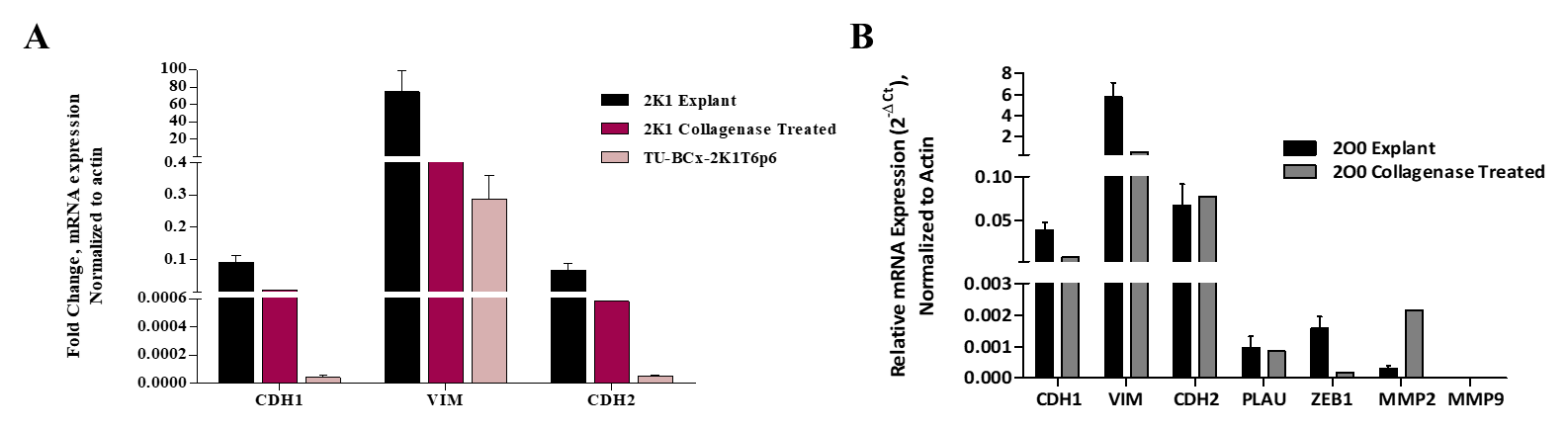
**

**Figure S6.** Comparison of genomic features of collagenase-treated TU-BcX-2K1 tumor to natural tumor and cell line-derived tumor. Analysis was performed using qRT-PCR. (A) Collagenase treatment resulted in less endogenous expression of CDH1, VIM and CDH2. This reduction was even more prominent in the cell line-derived tumor. (B) Similar results of collagenase-treated tumor compared to natural tumor were observed in another TNBC PDX model established in our laboratory, TU-BcX-2O0.
