# Supplementary material for: Drug resistance profiling of a new triple negative breast cancer patient-derived xenograft model
Source: BMC Cancer. 2019 Mar 7;19:205. doi: 10.1186/s12885-019-5401-2 (PMC6407287; doi:10.1186/s12885-019-5401-2)
Supplement: Supplementary file 8 — Figure S8. Selected compounds from the NCI oncology panel that were resistant to TU-BcX-2 K1 cells. Cyclophosphamide, uramustine, carmustine and lomustine are all alkylating agents. (DOCX 248 kb) [file 12885_2019_5401_MOESM8_ESM.docx]

**
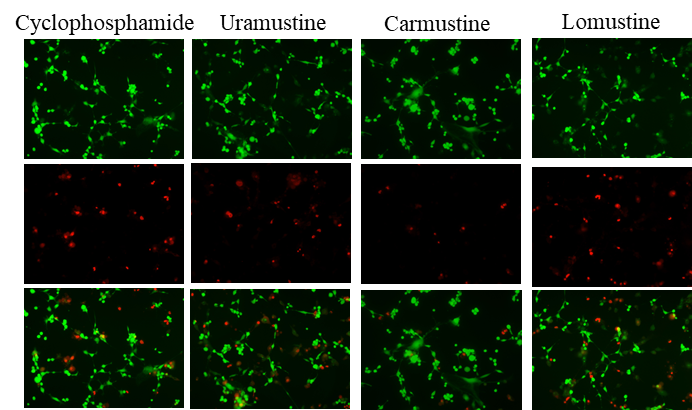
**

**Figure S8**. Selected compounds from the NCI oncology panel that were resistant to TU-BcX-2K1 cells. Cyclophosphamide, uramustine, carmustine and lomustine are all alkylating agents.
