# Supplementary material for: Drug resistance profiling of a new triple negative breast cancer patient-derived xenograft model
Source: BMC Cancer. 2019 Mar 7;19:205. doi: 10.1186/s12885-019-5401-2 (PMC6407287; doi:10.1186/s12885-019-5401-2)
Supplement: Supplementary file 9 — Figure S9. TU-BcX-2 K1 spheres were resistant to treatment with Taxol, independent of the dose used. (A) Adherent culture of TU-BcX-2 K1 cells treated with Taxol at varying doses (10 nM, 100 nM, 1 μM, 10 μM) for 72 h before fixed and stained with Crystal Violet. (B) Sphere culture of TU-BcX-2 K1 cells treated with Taxol at the same varying doses for 72 h before stained with the Live/Dead Kit. Live cells were stained by Calcein-AM (green) and dead cells were stained with EthD-III (red). N = 3 for all experiments. (DOCX 182 kb) [file 12885_2019_5401_MOESM9_ESM.docx]

**
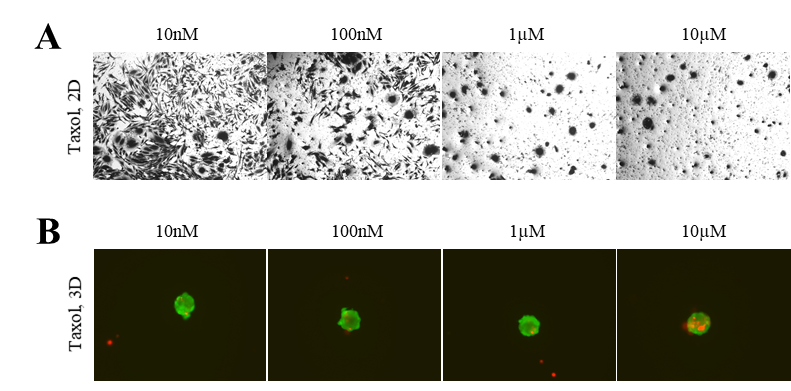
**

**Figure S9.** TU-BcX-2K1 spheres were resistant to treatment with Taxol, independent of the dose used. (A) Adherent culture of TU-BcX-2K1 cells treated with Taxol at varying doses (10nM, 100nM, 1µM, 10µM) for 72 hours before fixed and stained with Crystal Violet. (B) Sphere culture of TU-BcX-2K1 cells treated with Taxol at the same varying doses for 72 hours before stained with the Live/Dead Kit. Live cells were stained by Calcein-AM (green) and dead cells were stained with EthD-III (red). N = 3 for all experiments.
