# Supplementary material for: Drug resistance profiling of a new triple negative breast cancer patient-derived xenograft model
Source: BMC Cancer. 2019 Mar 7;19:205. doi: 10.1186/s12885-019-5401-2 (PMC6407287; doi:10.1186/s12885-019-5401-2)
Supplement: Supplementary file 10 — Figure S10. TU-BcX-2 K1 primary mammospheres’ response to selected cytotoxic oncology drugs. (A) TU-BCx-2 K1 explants were embedded in 40% Matrigel™ and immunofluorescence was employed to evaluate CD44 (red) and CD24 (green) populations within the spheres. ‘T’ indicates the tumor explant from which the mammospheres budded. (B) z-stack imaging of the explant-derived mammospheres reveals CD44high cells surround the mammosphere. (C) TU-BcX-2 K1 spheres embedded in Matrigel™ were pre-treated with DMSO (0.1%), Taxol (10 nM) and romidepsin (100 nM) for 72 h before immunofluorescence staining with CD44 (red), CD24 (green) and DAPI nuclear stain (blue). Representative images are shown. Images were captured at 50X magnification. (DOCX 381 kb) [file 12885_2019_5401_MOESM10_ESM.docx]

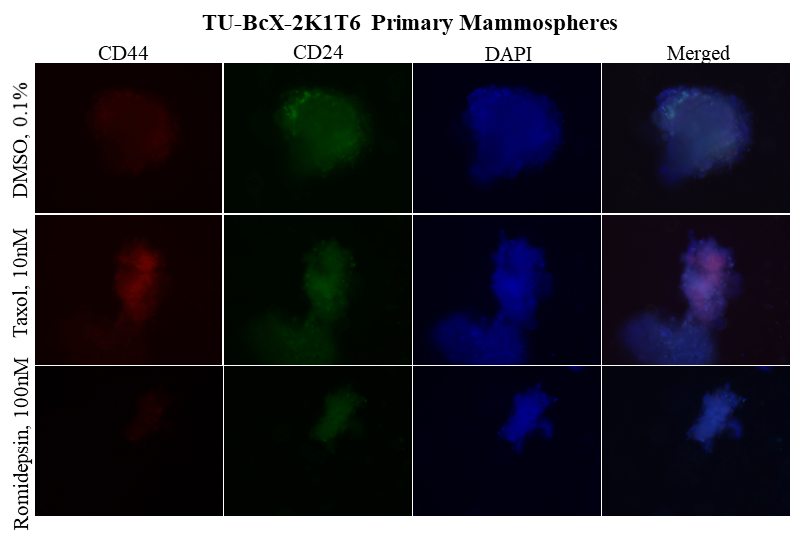


**Figure S10. TU-BcX-2K1 primary mammospheres’ response to selected cytotoxic oncology drugs.** TU-BcX-2K1 spheres embedded in Matrigel^TM^ were pre-treated with DMSO (0.1%), Taxol (10nM) and romidepsin (100nM) for 72 hours before immunofluorescence staining with CD44 (red), CD24 (green) and DAPI nuclear stain (blue). Representative images are shown. Images were captured at 50X magnification.
