# Supplementary material for: Drug resistance profiling of a new triple negative breast cancer patient-derived xenograft model
Source: BMC Cancer. 2019 Mar 7;19:205. doi: 10.1186/s12885-019-5401-2 (PMC6407287; doi:10.1186/s12885-019-5401-2)
Supplement: Supplementary file 11 — Figure S11. Morphology-related gene changes after TU-BcX-2 K1 explants were treated with panobinostat and romidepsin. Genes analyzed were the epithelial gene E-cadherin (CDH1) and the mesenchymal genes vimentin (VIM) and N-cadherin (CDH2). qRT-PCR was used to evaluate gene expression and results were normalized to β-actin and DMSO vehicle controls. Error bars represent S.E.M. N = 3. (DOCX 22 kb) [file 12885_2019_5401_MOESM11_ESM.docx]

**
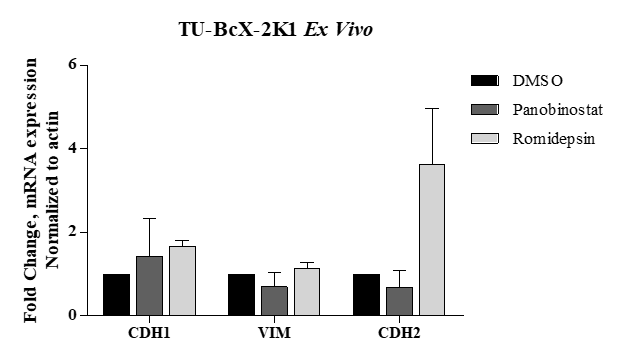
**

**Figure S11.** Morphology-related gene changes after TU-BcX-2K1 explants were treated with panobinostat and romidepsin. Genes analyzed were the epithelial gene E-cadherin (CDH1) and the mesenchymal genes vimentin (VIM) and N-cadherin (CDH2). qRT-PCR was used to evaluate gene expression and results were normalized to β-actin and DMSO vehicle controls. Error bars represent S.E.M. N = 3.
